# Massive-Scale RNA-Seq Analysis of Non Ribosomal Transcriptome in Human Trisomy 21

**DOI:** 10.1371/journal.pone.0018493

**Published:** 2011-04-20

**Authors:** Valerio Costa, Claudia Angelini, Luciana D'Apice, Margherita Mutarelli, Amelia Casamassimi, Linda Sommese, Maria Assunta Gallo, Marianna Aprile, Roberta Esposito, Luigi Leone, Aldo Donizetti, Stefania Crispi, Monica Rienzo, Berardo Sarubbi, Raffaele Calabrò, Marco Picardi, Paola Salvatore, Teresa Infante, Piergiuseppe De Berardinis, Claudio Napoli, Alfredo Ciccodicola

**Affiliations:** 1 Institute of Genetics and Biophysics “A. Buzzati-Traverso”, CNR, Naples, Italy; 2 Istituto per le Applicazioni del Calcolo, Mauro Picone, CNR, Naples, Italy; 3 Institute of Protein Biochemistry, CNR, Naples, Italy; 4 Telethon Institute of Genetics and Medicine (TIGEM), Naples, Italy; 5 Department of General Pathology and Excellence Research Centre on Cardiovascular Diseases, 1st School of Medicine, Second University of Naples, Naples, Italy; 6 Section of Microbiology, Department of Experimental Medicine, 1st School of Medicine, Second University of Naples, Naples, Italy; 7 Centro Diagnostico San Ciro, Portici, Italy; 8 Cardiology Department of Second University of Naples, “Monaldi Hospital”, Naples, Italy; 9 Department of Biochemistry and Medical Biotechnology, University of Naples “Federico II”, Naples, Italy; 10 Department of Cellular and Molecular Biology and Pathology “L. Califano”, University of Naples “Federico II” and Ceinge Biotecnologie Avanzate s.c.a.r.l., Naples, Italy; 11 Fondazione-SDN (Institute of Diagnostic and Nuclear Development), IRCCS, Naples, Italy; Fondazione Telethon, Italy

## Abstract

Hybridization- and tag-based technologies have been successfully used in Down syndrome to identify genes involved in various aspects of the pathogenesis. However, these technologies suffer from several limits and drawbacks and, to date, information about rare, even though relevant, RNA species such as long and small non-coding RNAs, is completely missing. Indeed, none of published works has still described the whole transcriptional landscape of Down syndrome. Although the recent advances in high-throughput RNA sequencing have revealed the complexity of transcriptomes, most of them rely on polyA enrichment protocols, able to detect only a small fraction of total RNA content. On the opposite end, massive-scale RNA sequencing on rRNA-depleted samples allows the survey of the complete set of coding and non-coding RNA species, now emerging as novel contributors to pathogenic mechanisms. Hence, in this work we analysed for the first time the complete transcriptome of human trisomic endothelial progenitor cells to an unprecedented level of resolution and sensitivity by RNA-sequencing. Our analysis allowed us to detect differential expression of even low expressed genes crucial for the pathogenesis, to disclose novel regions of active transcription outside yet annotated *loci*, and to investigate a plethora of non-polyadenilated long as well as short non coding RNAs. Novel splice isoforms for a large subset of crucial genes, and novel extended untranslated regions for known genes—possibly novel miRNA targets or regulatory sites for gene transcription—were also identified in this study. Coupling the rRNA depletion of samples, followed by high-throughput RNA-sequencing, to the easy availability of these cells renders this approach very feasible for transcriptome studies, offering the possibility of investigating in-depth blood-related pathological features of Down syndrome, as well as other genetic disorders.

## Introduction

Expression profiles of thousands of genes in various organs and cell lines have been successfully determined by using different methods and approaches such as microarray, serial and cap analysis of gene expression, and massively parallel signature sequencing [1]–[11]. These approaches have led to the identification of differentially expressed genes in physiological and pathological conditions, such as Down syndrome (DS) [12]–[15], Alzheimer, Parkinson [16]–[18] and cardiovascular diseases [2], [8], [19], [20].

In Down syndrome the dosage imbalance of human chromosome 21 (HSA21) genes, and the subsequent global gene deregulation observed overall the genome [12], [21], [22], have long been associated to different aspects of DS pathogenesis. Expression analyses of DS tissues and mouse models have reported conflicting results [23], [24], showing that HSA21 gene expression greatly varies across trisomic tissues [14], [22]. However, most of published works has focused on hybridization-based technologies - suffering from hybridization and cross-hybridization artefacts and offering a limited dynamic range - or tag-based approaches, suffering for the ambiguous mapping of their short reads. Hence, to date we completely lack information about other rare, even though physiologically relevant, RNA classes such as small coding and (long-) non-coding RNAs. In addition, there is not yet evidence of DS-specific splice isoforms for genes crucial in the pathogenesis and, to date, none of published works has described, in a single experiment, the complete transcriptional networks in Down syndrome.

The introduction of next generation sequencing (NGS) technologies has revealed the complexity of mammalian transcriptomes, enabling to effectively explore - with an unprecedented throughput capacity - simple and complex genomes [25]–[31]. NGS have shown that most of nucleotides are expressed, highlighting that only a small fraction of all transcribed sequences (less than 2%) is represented by mRNA [32], [33], and that not yet well-characterized RNA species, such as microRNA recently described in DS [34] as well as small nucleolar RNA (snoRNA), are emerging as potential factors contributing to pathological phenotypes [35], [36].

In the last years, in order to identify genes contributing to DS phenotype and to its phenotypic variability, the above-mentioned standard approaches for gene expression profiling have been applied to several mouse models with segmental duplications of DNA segments orthologous to human chromosome 21. Alternatively, transcriptome studies on human DS subjects have been so far performed on *post-mortem* tissues and/or fetuses, and few studies have focused on RNAs isolated from human adult whole blood samples [12], [37]–[39]. Thus, it would be clinically relevant to investigate, with an innovative and high-throughput approach, early gene regulatory mechanisms linked to cardiovascular disease, cancer and immune disorders linked to DS.

To this purpose, we analysed for the first time the global transcriptome of human trisomic and euploid endothelial progenitor cells (EPCs) to an unprecedented level of resolution and sensitivity by RNA-Seq on a next generation sequencing platform. By using a selective depletion of abundant rRNA molecules from samples - followed by the sequencing of strand-specific cDNA libraries - we were able to measure the effects of trisomy 21 in a specific cell type affected in DS, and also to quantify the defect during postnatal development, possibly correlating gene expression changes to the observed phenotype. Indeed, literature data and our recent findings strongly indicate that circulating EPCs, whose levels are linked to tissue regeneration, are impaired in DS [12], [40]–[42]. These cells play pivotal role in the maintenance of endothelium integrity, repair after injury and postnatal neovascularization and several studies suggest their use in the clinical setting [43]–[47]. Moreover, accumulating evidences indicate a reduced availability, and/or impaired EPC function in cardiovascular and metabolic diseases [12], [45], [48]–[50]. Endothelial dysfunction, angiogenesis suppression and infection recurrence are hallmarks of DS, and the impairment in the number and function of circulating progenitors may promote a wide number of diseases. The massive-scale RNA-Seq and the easy availability of these cells from affected individuals allow to shed light on endothelium-related pathological features of DS, rendering this analysis feasible on a large number of samples.

## Results

### Strand-oriented libraries preparation and sequencing

The ability, and the power, to measure gene expression in RNA-Seq experiments is strictly correlated to the number of sequence reads mapped to transcribed regions in a particular cell/tissue/organism. In the light of this, for a whole-transcriptome (WT) analysis we planned both our sequencing strategy and platform usage (Figure S1).

To this aim, a systematic depletion - from total RNA samples - of very abundant rRNA molecules (consisting of about 95% of cellular RNA), was performed. This procedure, coupled with the massive sequencing on NGS platform, allows to investigate the entire transcriptional landscape of an organism, offering the possibility to analyse - within the same experiment - polyA^+^ mRNAs, long as well as small coding and non-coding RNA species. It clearly represents a great opportunity, and a challenge, compared to the commonly used approaches relying on polyA^+^ enrichment of the samples [30]–[32], [51]–[57].

In addition, since preserving the strandedness is fundamental for further data analysis and interpretation, we created strand-oriented libraries (SOLs) for each sample. Indeed, SOLs usage allows to determine the correct directionality of transcription and gene orientation (for both annotated and unannotated expressed regions), thus facilitating the detection of opposing and overlapping transcripts.

In this study, we generated SOLs from rRNA-depleted total RNAs isolated from human EPCs [7], [58] of a female affected from trisomy 21 and one age- and sex-matched euploid, and sequenced them to a depth of about 100 million of 50 nt reads per library on a SOLiD v3 platform (Applied Biosystems).

### Mapping strategy and visualization

The sequenced reads were mapped on human genome (hg19) using RNA-MATE [30]. Mapping strategy and results are illustrated in Figure S2 and Table S1. Details of the mapping strategy are given in “Materials and Methods” and File S1.

We noted that filtering reads derived from very abundant rRNAs molecules (5.8S, 18S, 28S) has a great impact on rRNA-depleted WT experiments since they still constitute a significant fraction of total sequenced reads, whereas adapter-filtered reads represent a negligible amount. However, at least for the purpose of this work, they can be used as a measure of ribodepletion efficiency rather than a real measure of interest.

The cyclic alignment implemented in RNA-MATE ensured the detection of expressed regions from both annotated exons and junctions from a custom library, also giving the possibility to detect the expression of previously unannotated regions and to identify novel combinatorial exon usage for every known *locus*. The low extent of antisense mapping of reads (about 0.07% for both libraries) to splice junctions' libraries, was used to assess SOLs' directionality and to tune the mapping parameters. In addition, most of reads (about 90%) that mapped to the genome and to junction library were 50 nt in length with few sequence mismatches. Such results are comparable to those obtained in analogous studies and constitute an overall measure of the quality the produced data.

At the end of the alignment strategy three types of reads were distinguished: uniquely assignable reads (UARs), multiple reads (MRs) and reads without a specific mapping location (denoted as unmatched reads; see “Materials and Methods”, Figure S2 and Figure S3). For the sake of simplicity we considered only UARs and reads mapping on junction library for further analyses. We noted that discarding MRs - which mainly derive from conserved domains of gene families and/or common repeats - is likely to introduce an experimental bias, decreasing the coverage and reducing the possibility to investigate expressed retrotransposons and most of highly conserved gene families [26]. However, since a significant fraction of multiple reads was assigned to UARs category using a rescue procedure, we reduced the above-mentioned mapping bias (“Materials and Methods” and File S1) [59].

We also noted that the sequenced reads mapped at 50 nt length (with few mismatches) contribute to about 91% of DS and 86% of euploid unique reads, and to about 76% and 71% of finally assigned UARs for DS and euploid, respectively.

We observed, as expected due to the presence of an extra copy of HSA21 for DS sample, a higher amount of sequenced reads mapping to this chromosome, with the highest (1.33) DS/euploid mapped reads ratio than observed for the other chromosomes (mean ratio 0.99±0.05). A similar unbalancing in reads' mapping was also observed for the mitochondrial chromosome (chr M) (ratio = 1.23) mainly due to the highly variable number of mitochondria in a cell, organism and tissue type.

To better elucidate the landscape of gene expression in both states we classified all mapped reads in the following categories: reads mapping to 1) annotated RefSeq gene models, 2) intronic regions, 3) intergenic regions, 4) known RefSeq splice junctions and 5) novel combinatorial junctions and 6) mitochondrial genome. The mapping of the categories from 1) to 3) and 6) is depicted in Figure S4. The analysis of each category is described in the following sections.

### Gene expression quantification

Since we previously described in EPCs isolated from DS individuals a global deregualtion of gene expression compared to euploid cells [14], we used RNA-Seq to have a better quantitative estimate of gene expression from both known genes and previously uncharacterized expressed regions. To this aim we scored each *locus* activity in both trisomic and euploid cells by counting the number of reads mapping to annotated RefSeq transcripts (release 38) [60]. In particular, for gene *loci* with a single transcript we estimated gene expression as the number of UARs mapping to the entire length of the transcript, whilst for genes with multiple splice isoforms a measure of global *locus* activity was obtained summing the reads mapped to any independent exon (or part of exon) of each possible transcript (details in File S1). In both cases, the reads count deriving from reads mapped to the junctions library were added to each corresponding *locus*.

**Figure 1:**
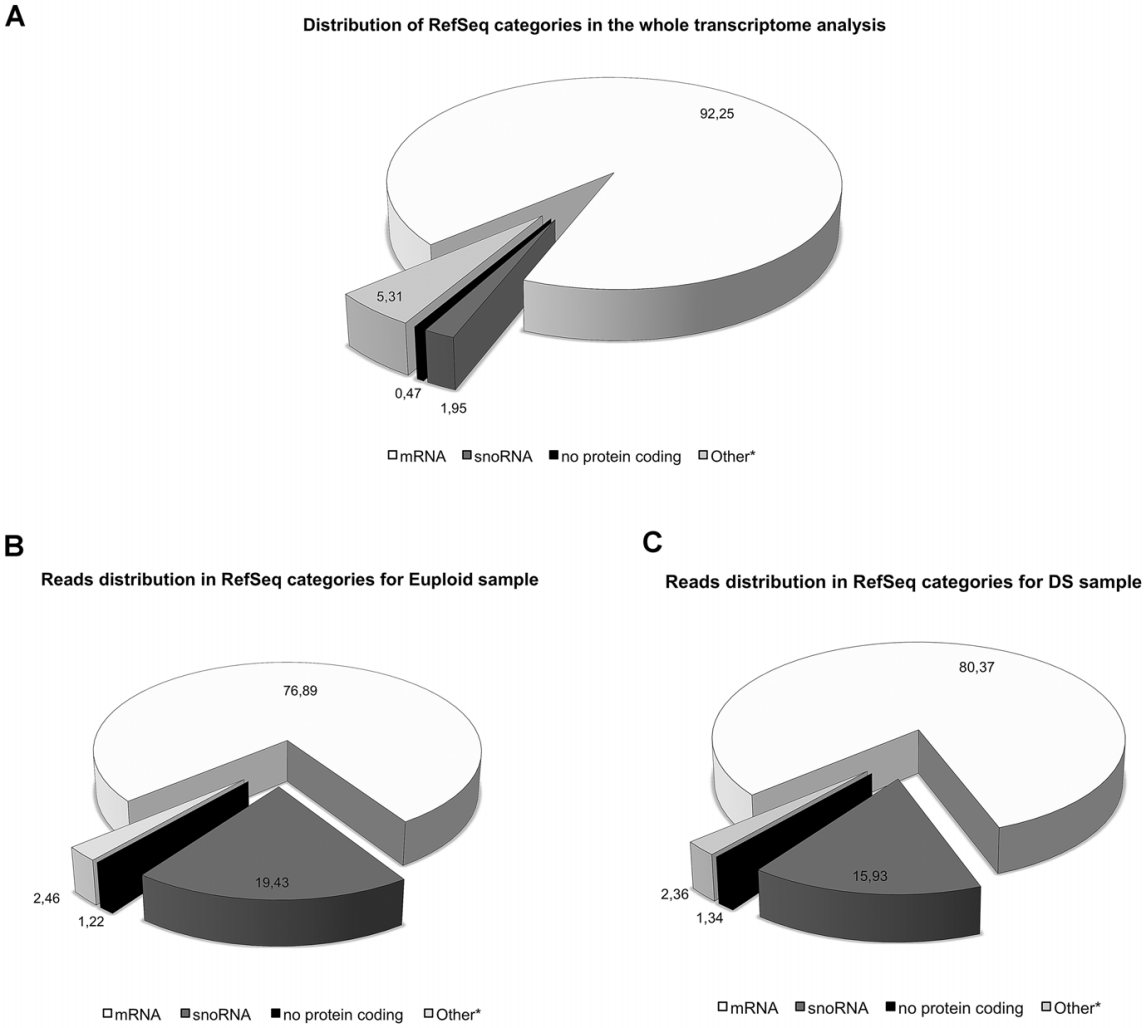
RefSeq categories and reads distribution. Distribution of the abundance of the RefSeq categories (HGNC) in the observed actively transcribed loci of the two states (A); Distribution of the UARs across the distinct RefSeq categories. DS (B) and euploid (C). The “other” category, marked with asterisk, include less represented RNAs (pseudogenes, microRNA, snRNA, scRNA, antisense, vault and RNAse) according to HGNC. Percentages are shown in the pie chart.

The representative RefSeq categories (Human Gene Nomenclature Committee, HGNC) [61] comprising all the genes detected and analyzed in the WT experiment are shown in Figure 1A. In DS as well as euploid sample, about 92% of detected *loci* with evidence of active transcription in circulating progenitors fall in the mRNA category. Surprisingly, the distribution of mapped reads per category revealed a 2- to 10-fold enrichment of non-coding RNAs, particularly snoRNA, in both analyzed samples (Figure 1B and 1C). Moreover, the distribution of mapped reads (in terms of genomic positions) showed, as expected, a strong bias toward regions already annotated as genes in RefSeq: on average, about 50% of mapped reads fell in such regions. However, we noted that such percentage is significantly smaller than observed using polyA^+^ enrichment protocols.

The gene expression values of already annotated genes were measured and expressed as reads per kilobase of transcript (or gene model) per million mapped reads (RPKM) [55]. Using a threshold of 0.1 RPKM, we detected a total of 17474 and 16800 RefSeq genes for DS and euploid EPCs respectively with at least one mapped read, and 13144 RefSeq genes with evidence of active transcription common for both trisomic and euploid EPCs.

In particular, due to our interest in investigating gene expression in the context of DS, we also focused on HSA21 genes. Hence, on a total of 260 RefSeq annotated HSA21 genes, we detected 148 and 141 genes expressed at levels below the threshold for DS and euploid EPCs samples, respectively.

**Figure 2:**
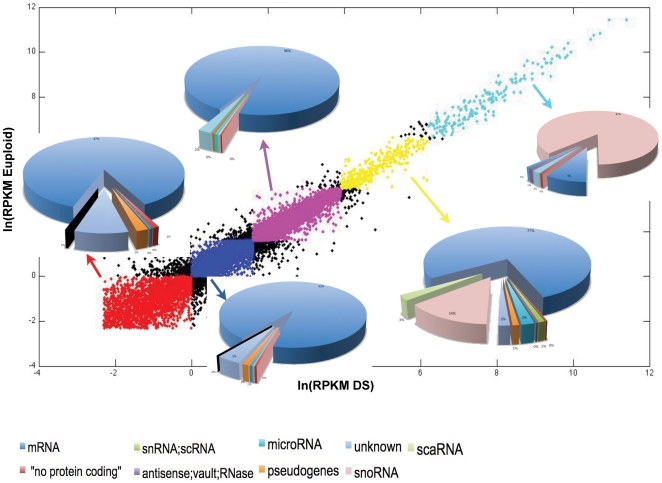
Comparison of RPKM content for RefSeq genes. Distribution of RefSeq categories (according to HGNC) within each class of RPKM.

All RefSeq genes, whose expression was detected within the experiment, were further classified according to RPKM values in 5 categories of expression: 1) very low, 2) low, 3) intermediate, 4) high and 5) very high (Figure 2; see “Materials and Methods”). This categorization revealed us, for both trisomic and euploid samples, a strong enrichment of snoRNAs in the highest RPKM categories (these RNAs were about 15% of total genes in category 4, and about 90% in category 5), clearly showing these molecules are below mRNAs – and if we exclude rRNAs - the second RNA group for abundance, and they also represent the more expressed RNA fraction in rRNA-depleted WT experiment.

To visualize in a user-friendly way the gene expression data derived from reads mapping, we prepared genome-wide, strand-specific, nucleotide-resolution files for each library corresponding to the trisomic and euploid states. In particular, these files contain information about reads mapping to the entire human genome, to splice junctions and RPKM categories for each analyzed RefSeq gene (see File S1). These resources represent a very powerful tool for genetics and genomics studies as they allow to easily investigate the entire landscape of gene expression alongside public genome annotations within UCSC Genome Browser [62] as “custom tracks” (Supplementary files available upon request).

### Evidence and quantification of intronic and intergenic transcription

An intriguing finding of this study was the observation that, in both DS and euploid libraries, about 50% of all mapped reads occurred outside the annotated *loci*, outside the furthest 5′ and 3′ exons of already known genes, strongly indicating that many RefSeq genes may require extension or revision. This finding also suggests that this relevant extent of extra-genic transcription may possibly account for some of the pathological features observed in Down syndrome, as well as it is likely to occur for other human inherited disorders.

Thus, to address the extent of intronic and intergenic transcription, reads mapping to hg19 in non-RefSeq regions were divided into three categories: 1) intronic (inR) and 2) intergenic (igR) regions, and 3) chr M.

In particular, in the trisomic sample, about 8.7 M of sequenced reads mapped to inRs, 5.9 M into igRs and 2.0 M to chrM, for a total of 16.6 M of reads mapped to non-RefSeq regions. For the euploid sample, about 7.3 M of reads mapped to inRs, 5.6 M into igRs and 1.4 M to chrM for a total of 14.3 M of reads mapped to non-RefSeq regions (Figure S4).

To identify yet unannotated transcribed regions, potentially representing novel disease-specific expressed regions, and to better elucidate the still uncharacterized landscape of gene expression in trisomic EPCs compared to euploid cells, reads were further filtered with combined annotations from UCSC “known genes” and Ensembl databases (File S1) [63], [64]. We found that in DS sample about 4.6 M of reads (of which 2 M from chr M and 2.6 M from both intergenic and intronic) were supporting either UCSC or Ensembl annotation, whilst more interestingly 12 M of reads were still mapped to unannotated regions. In the euploid sample, about 3.9 M (of which 1.4 M from chr M and 2.5 M from both intergenic and intronic) supported either the annotations, whilst 10.4 M of reads still mapped to unannotated regions. We also observed that most UCSC and Ensembl annotations covered about 98% of the reads mapping on chr M, about 30% of intergenic and about 10% of intronic regions, for both samples.

Finally, the reads mapping to yet unannotated regions, from both DS and euploid samples were pooled together and used to predict candidate novel intronic and intergenic transcriptionally active regions (inTARs and igTARs, respectively) - possibly representing new genes - or to revise previously annotated gene models (definitions are given in File S1).

To this purpose, we noticed that 4.2 M of mapped reads in DS and 3.9 M in euploid - assigned to intergenic unannotated regions - spanned across 4.9×10^9^ bp (considering both strands), and 7.8 M of mapped reads in DS and 6.5 M in euploid - assigned to unannotated intronic regions - spanned across 1×10^9^ bp (considering both strands). The size of, such huge, unannotated regions do not allow to easily identify the presence of significant signal (i.e density of reads mapping together) from the background noise, resembling the search of a needle in a haystack. Therefore, *ad hoc* refinement procedure with **W** = 500 and **T** = 30 (described in “Refinement of non-RefSeq loci”) was used to automatically extract reads' dense transcriptionally active regions in a computationally fast way. The refinement procedure, applied on the pooled samples, allowed us to define 21804 igTARs (spanning across about 17 Mb) in which for both samples mapped about 1.8 M of sequenced reads (about 45% of unannotated intergenic reads). In a similar way, we defined 99030 inTARs (spanning across about 80 Mb) in which were mapped about 4.1 M and 3.7 M of reads for DS and euploid, respectively (more than 55% of unannotated intronic reads for both samples). All regions were annotated in a BED format and the expression levels of both inTARs and igTARs were then measured for each sample.

Since not yet annotated TARs may be relevant for DS pathogenesis, we focused on the quantitative evaluation of these regions. The analysis revealed that 21648 igTARS and 98156 inTARs were transcriptionally active in DS progenitor cells, whereas 21608 igTARS and 97709 inTARs were active in the euploid state. Of these, 21460 igTARs were regions of active transcription common to both states, whilst 187 and 148 were respectively DS and euploid specific. Similarly, we identified 96864 inTARs common to both samples, with 1092 and 745 regions DS and euploid specific, respectively.

A random selection of a small subset of newly identified TARs underwent manual curation for further analysis. Particularly, we noted that many highly expressed unannotated regions felt in large repeats family (RepeatMasker based on RepBase library), comprising short - which include *Alu* family - and long interspersed nuclear elements (SINE and LINE), spanning overall the human genome, and also RNA repeats (such as SSU-rRNA family). However, an accurate estimate of the expression within such regions is strongly biased in both samples due to the multiple localization of these regions alongside the human genome, and thus further focused studies are needed in order to better address the extent of expression of such repeats families.

In addition, we also scanned a subset of inTARs and igTARs for the presence of putative open reading frames (ORFs). The analysis revealed that a high fraction of these newly identified TARs, both intronic and intergenic contain ORFs longest than 200 bp. In particular, some inTARs conserved the correct frame of the gene they are located within, suggesting these are likely to represent alternative exons. On the other hand, it has been observed that a subset of analyzed igTARs (150–250 bp in length) did not show any ORF, suggesting they may represent novel small and long non-coding RNAs.

However, these preliminary findings strongly suggest these newly identified regions of active transcription require both further experimental validations - and also computational efforts - in order to address in a genome-wide fashion whether they represent novel genes - and/or exons of already known genes- and novel short (or long) intergenic transcripts, and whether the differential expression of these expressed extragenic regions may be linked at some extent to observed DS phenotypes.

**Figure 3:**
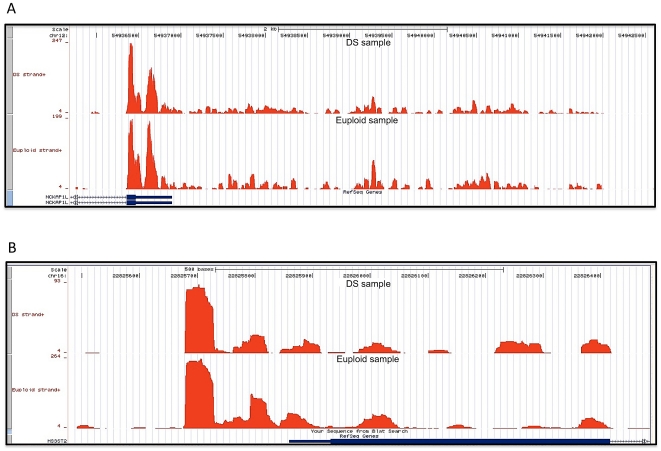
Evidence of 3′ and 5′ UTRs gene extensions. Illustration of 3′ (A) and 5′ (B) extended UTRs that are present in both samples.

Independently, we also studied the transcriptional activity in close proximity to 3′ and 5′ UTRs of RefSeq loci, in order to understand whether these regions could possibly represent extensions of already annotated genes. Particularly, we focused on expressed regions using a user-defined window 150 bp in length, located both upstream 5′ UTRs and downstream 3′ UTRs. We found 3600 and 2948 candidate genes showing a clear evidence of an extended 3′ UTR in DS and euploid samples, respectively (example in Figure 3A). Of these, 1868 extended regions were common to both samples, giving a strongest evidence for the refinement of untranslated regions of these RefSeq loci. We believe that state-specific extended UTRs (specifically those expressed in DS progenitor cells) may be important for gene expression regulation and/or for mRNA stability and processing, possibly accounting for some DS pathological features. More interestingly, we observed that most of newly-defined or extended 3′UTRs contain putative novel miRNA binding sites (data not shown), characteristic of 3′UTRs of annotated transcripts, suggesting these regions may potentially contribute to microRNA-mediated regulation of these transcripts. This finding is crucial for understanding putative novel mechanisms of regulation for genes already known to be involved in DS pathogenesis, and may also be helpful to identify novel candidates in the trisomy 21.

Finally, for 5′ UTRs we found a lower number of candidate genes (1491 and 1280 for DS and euploid, respectively) possibly needing annotation revision, which indicates that current annotations are more biased toward the 3′ UTRs of expressed transcripts (example in Figure 3B).

### Survey on the alternative splicing

Massive-scale RNA sequencing data, other than identifying differential expression of genes in a disease, are useful to human genetics in what they can be used to investigate alternative splicing, also discovering novel splice isoforms for crucial genes. For instance, identifying sequence reads that span exon-exon junctions could help to define exon usage and alternative splicing (although reconstructing entire transcripts will be challenging, particularly with short reads and it will require a very high coverage and the use of paired-end reads to achieve a good accuracy).

However, to illustrate the great potential of these data for studying both canonical and alternative splicing in the context of Down syndrome, we performed a preliminary analysis to identify reads that span exon-exon junctions. We detected a total of 92939 splice junctions in DS sample and 80200 in euploid; of these, 64115 and 56621 (DS and euploid, respectively) mapped with at least 3 sequenced reads, whilst 48604 and 43308 (DS and euploid, respectively) mapped with at least 5 sequenced reads (Table S2).

In addition, as expected for large-scale RNA-Seq data, we found evidence of several alternative splicing events (ASEs) in known RefSeq genes with a user-defined threshold of 3 and 5 mapped reads. To achieve a highest reliability of these data, we considered a user-defined threshold of at least 5 mapped reads as informative for ASEs (Figure S5). By using this approach, we found that 1621 splice junctions in DS and 1783 in euploid were representative of ASEs (i.e either multiple donor or multiple acceptor junctions; details in “Materials and Methods”).

**Figure 4:**
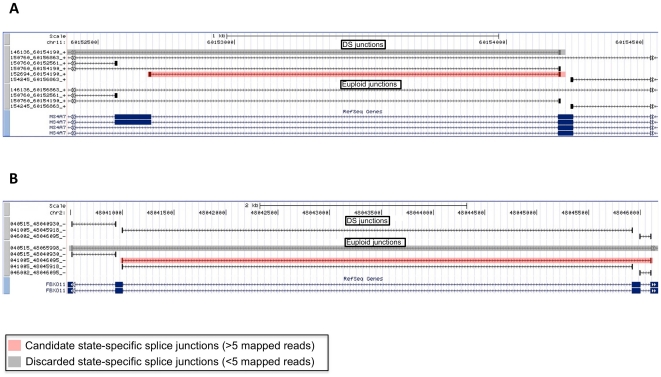
State-specific alternative splicing. Example of sample-specific alternative splicing events with T1 = 5. Reliable junctions are highlighted in light red for both cases (DS in panel A and euploid in panel B). Junctions highlighted in light grey are below the threshold. State-specific junctions are those not showing any hit in the other sample.

In order to identify ASEs specific of DS progenitor cells, avoiding a “threshold-dependent” exclusion of any given junction (i.e of junctions with a number of mapped reads slightly below the chosen threshold), we marked as “sample-specific” only junctions without any mapping in the euploid state (and *viceversa*). By using this procedure, we found that about 18% of all ASEs detected in each sample were sample specific. Indeed, we identified 294 DS-specific and 323 euploid-specific alternative splice events (Figure S5 and Table S2). Of these, 135 junctions for DS and 229 for euploid (45.9% and 70.9% of total state-specific ASEs, respectively) were completely unannotated (i.e. non-RefSeq, -UCSC, or -Ensembl), thus representing good candidates for further analyses aimed to fully characterize novel disease-specific isoforms within DS isolated EPCs. Examples of genes with evidence of sample specific splicing are depicted in Figure 4.

Interestingly, this analysis showed evidence of a DS-specific splice junction (transcript variant NM_130436.2; proteinID Q13627-2) in a crucial HSA21 gene involved in DS pathogenesis, namely *DYRK1A* [65]. For all mentioned cases, sequence reads supporting evidence of alternative splicing will be helpful for further detailed analyses aimed to resolve, if any, possible exon-annotation conflicts.

In the context of the syndrome, we also observed that some interesting genes, involved in the immune response and angiogenesis pathways - and previously shown to be deregulated in DS EPCs [12] - had evidence of yet unannotated sample-specific isoforms. Further analyses are needed to address whether these isoforms may play a role in DS pathogenesis.

### The transcriptome complexity of DS beyond the rRNA

NGS has revealed the evidence of previously not well, characterized - or completely uncharacterized - RNA molecules, emerging as crucial regulators of many biological processes and for their potential link to human diseases. Small RNAs, including miRNAs, regulators of gene expression involved in various cellular processes, as well as small nucleolar RNAs (snoRNAs) - central to ribosome maturation and guides for site-specific modification of rRNAs - are acquiring greater attention for their involvement in human inherited disorders [35], [36]. In addition, long as well as short non coding RNAs, whose functional significance is still debated, and other classes of coding and non coding RNAs have been also described at transcriptional start sites, splice sites or in large intergenic regions [66]–[68].

In our experiment, not limited to the annotated polyA^+^ mRNA fraction, we detected and quantified active transcription in both human trisomic and euploid isolated EPCs from snoRNAs, small nuclear RNA (snRNA), miRNAs and other non-coding RNA, including lincRNAs.

In particular, we focused on UARs mapping to annotated snoRNAs, for which, as above described for the RefSeq genes, we measured gene expression as RPKM. Evidence of active transcription from 289 snoRNA (171 C/D box snoRNAs alias SNORD genes, 95 H/ACA box snoRNAs alias SNORA genes and 23 Cajal body-specific scaRNAs) was observed for DS cells, and the expression of 289 snoRNAs (173 C/D box, 93 H/ACA box and 23 Cajal body-specific scaRNAs) was detected in the euploid state. For both analysed samples, we observed a significantly strong increase (about 170-fold) in mean RPKM values for this class of RNAs compared to ploy-A^+^ transcripts (Figure 2).

**Figure 5:**
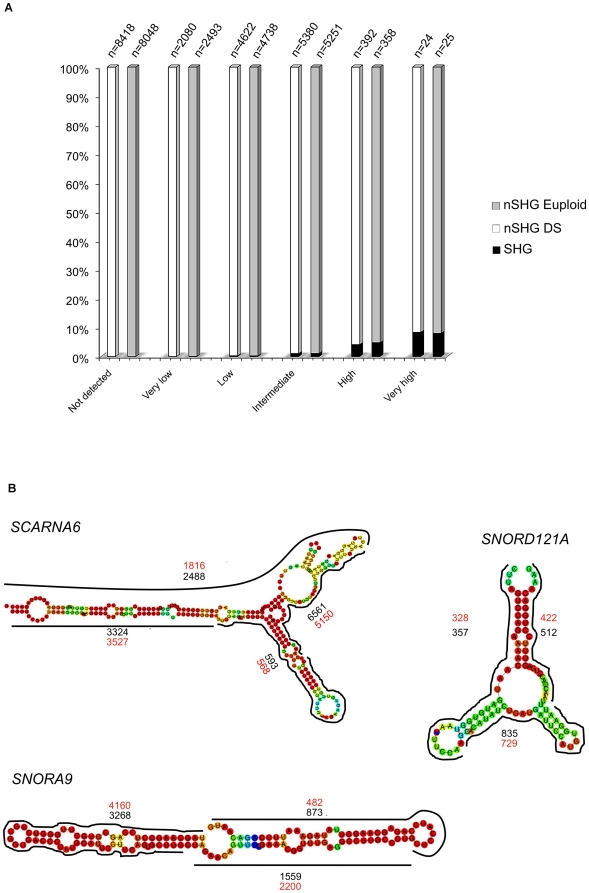
snoRNAs expression and mapping block patterns. (A) illustrates the percentage of snoRNAs host genes (SHG) vs non host genes (nSHG) within each RPKM category for both DS and euploid samples. (B) is a schematic representation of maximum coverage of few examples of snoRNAs, showing a characteristic mapping block pattern. Black and red numbers refer to DS and euploid maximum coverage, respectively.

In addition, we independently selected snoRNA belonging to “Very high” and “High” RPKM categories, which represent almost the totality of snoRNAs, and observed that the vast majority of these localize within the introns of RefSeq genes (namely host genes). Then, we analyzed the expression level, in terms of RPKM, of their related host genes. Table S3 shows the occurrence of each RPKM category of the host genes for two classes of snoRNAs, both in DS and euploid samples. We noted that 221 highly-expressed snoRNAs common to both states (76% of the total), preferentially - if not exclusively - mapped within intronic regions of highly-expressed genes (Figure 5A). More interestingly, none of highly-expressed snoRNA localized within introns or in close proximity of RefSeq genes with low or without any evidence of expression (Table S3), suggesting this class of small RNAs is preferentially located within euchromatic regions of very active transcription.

Moreover, since it has been recently shown that snoRNAs can be processed into snoRNA-derived RNAs (sdRNAs) [67], we analysed our WT data to address these specific features. Hence, we interestingly observed, as recently shown by Langenberger and colleagues [69], a correlation between reads' mapping pattern and snoRNAs processing steps, and possibly with their structural - and thus functional - properties (Figure 5B). As there depicted, snoRNAs clearly show specific block patterns with a characteristic reads coverage distribution. The particular enrichment of reads mapping to specific snoRNA sites is very likely to be correlated to its processing steps. A similar correlation with the secondary structure processing of non coding RNAs, even though at a lower extent due to RNA extraction protocol and library construction, was also observed for miRNAs (data not shown). However, these findings highlight the great potential of RNA-Seq data, deriving from ribosomal RNA-depleted samples rather than polyA^+^ enrichment procedure, for a better functional classification and the identification of novel non-coding RNAs.

Furthermore, a significant differential expression (DE) of snoRNAs in human trisomic EPCs (compared to euploid) was also observed (Table S4). In particular, 46 C/D box snoRNAs (3 up- and 43 down-regulated), 31 H/ACA box (9 up- and 22 down-regulated) and 9 Cajal body-specific scaRNAs (2 up- and 7 down-regulated) were differentially expressed in DS compared to euploid cells. Interestingly, we noted that the gene with the highest expression of HSA21 was a member of H/ACA box, *SNORA80*, which showed a strong evidence of DE in the trisomic cells.

Similarly, the expression of annotated RefSeq miRNA encoding genes was also measured. Expression from about 180 of them was detected, although about 20% of them had a small number of mapped reads in both samples. A significant DE in DS isolated progenitors compared to euploid, was also observed for a small subset of them (15 miRNA with a significant number of mapped reads; data not shown).

Finally, we also measured the expression from annotated lincRNAs (*Homo sapiens* GRCh37, Ensembl 58). Since the average length of these regions was significantly higher than RefSeq genes, on average RPKM values were smaller. However, a significant expression was detected for 1335 and 1269 regions for DS and euploid sample respectively, even though a subset of them completely or partially overlapped with RefSeq genes or repeated regions. After removing them from further analyses, we observed a significant differential expression in the trisomic state for 45 lincRNAs (Figure S6).

### Differentially expressed genes in human trisomy 21

Given the quantitative nature of our analysis, we used UARs and reads mapping to junction library to detect DE genes between the trisomic and euploid states.

In particular, we observed 1629 DE genes marked as “good” (about 12% of total detected genes in both samples), 158 as “strong”, 54 “acceptable”, whilst a large fraction (1827 genes) showed weak evidence of DE in the trisomic state since it did not pass the 1.5 fold-change cut-off. We selected only DE genes marked “strong” and “good” for further analyses (definitions are given in “Materials and Methods”).

**Figure 6:**
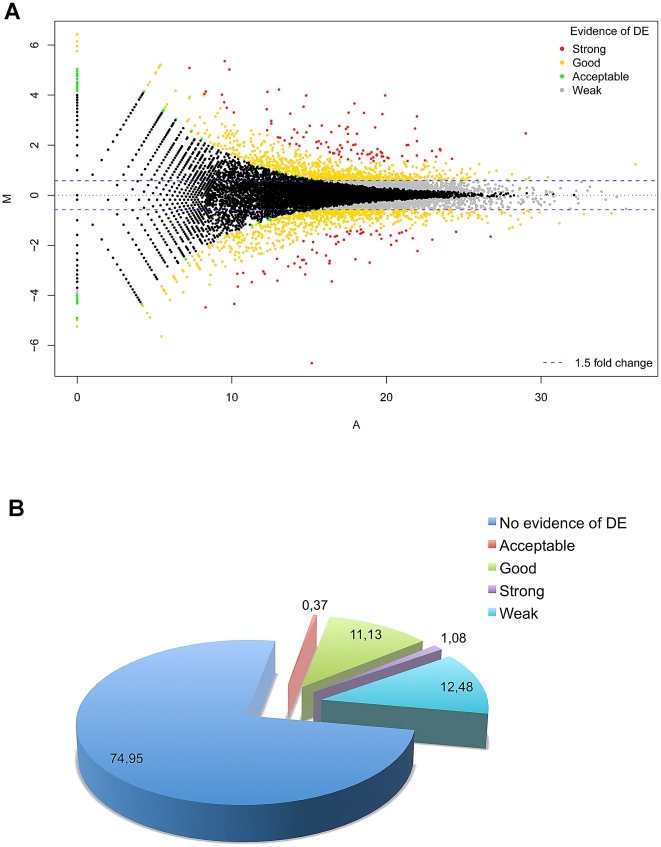
Differentially expressed RefSeq genes in human trisomy 21. (A) Standard MA-plot of the normalized global observed counts per each RefSeq gene. (B) shows the percentage of RefSeq genes classified as strong, good, acceptable evidence of DE with respect to those not showing any statistical evidence.

Of these 1787 genes showing evidence of differential expression between samples, 956 were up-regulated and 831 down-regulated in DS endothelial progenitors (Figure 6A). In contrast, about 75% of RefSeq annotated genes did not show any evidence of DE in the syndrome (Figure 6B). We also observed that 55 HSA21 genes - out of the 132 expressed in both DS and euploid cells - were DE in the trisomic state and, more interestingly, most of them (50 genes out of 55 HSA21 genes differentially expressed in DS) were up-regulated. Quantitative Real-Time PCR was used to validate the expression values in 24 actively transcribed *loci* per sample, confirming the evidence of DE also for genes marked as “weak” or “no change” (Figure S7 and Table S5).

**Figure 7:**
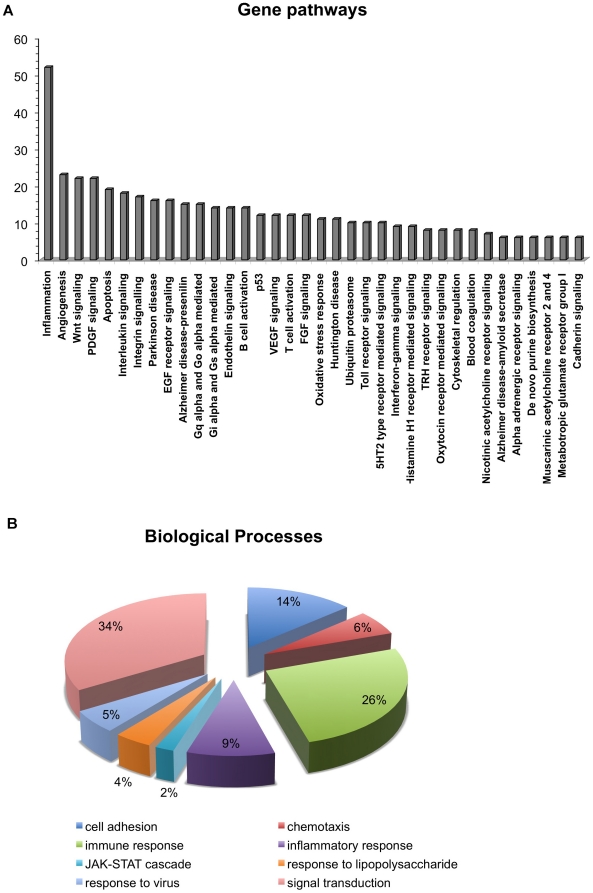
Pathway of differentially expressed RefSeq genes in DS sample. Bar graph representation of differentially expressed genes in DS vs euploid samples. (A) More enriched gene pathways are represented. The number of total DE RefSeq genes is also depicted. (B) Pie chart showing the percentages of representative GO terms (biological processes) enriched in DE genes in the DS sample compared to euploid.

The list of DE genes was then analyzed by using PANTHER (Protein ANalysis THrough Evolutionary Relationships) Classification System [70] in order to establish the occurrence of more representative deregulated pathways in the syndrome. The analysis revealed a particular enrichment for inflammation, angiogenesis, integrin and Wnt signaling pathways (Figure 7A). In addition, to highlight the most relevant biological processes possibly contributing to DS phenotypes previously observed in EPCs [12], we used a newly developed application for Gene Ontology (GO) analysis on RNA-seq data, namely GO-Seq [71]. By using the selection of genes DE within DS progenitor cells, we observed a particular enrichment for GO terms related to immune and inflammatory responses, cell adhesion and chemokine/cytokine receptor activities (Figure 7B). These GO terms are in agreement with the independent analysis of enriched gene pathways performed with PANTHER. Taken together these findings, which confirm independent results deriving from a genome-wide microarray analysis on EPCs isolated from young DS [12], strongly suggest that these biological processes, and the related genes, require much attention to further address their involvement in DS vascular and immune-related phenotypes.

Furthermore, in order to understand whether the newly identified igTARs and inTARs were differentially expressed within the syndrome, a similar approach was also used. In particular, we found that 44, out of the total 21804 igTARs identified in DS cells, were classified as strong DE regions, 1792 showed good evidence of DE, and 130 were classified as acceptable DE. For what concerns the inTARs, among the 99030 defined regions, we found 48 of them with strong evidences of DE in DS sample, 3173 with good and 720 defined as acceptable evidence of DE. In both cases, we noticed that the observed fold changes were sufficiently large, hence the threshold effect was negligible. Results are shown in Figure S8. These results suggest a possible involvement of such expressed regions in the pathogenesis of this syndrome, indicating that some yet unknown genetic determinants may be responsible of, or contribute to, the wide spectrum of DS pathological phenotypes.

## Discussion

RNA-Seq experiments revealed that the transcriptional landscape in higher eukaryotes is much more complex than previously anticipated, with a high proportion of transcripts originating from intergenic regions, referred to as “dark matter” [72], [73], thought to be transcriptionally silent or antisense to genes [33]. Previously published transcriptome sequencing data based on the polyA^+^ enrichment started to shed light on the transcriptional complexity in humans and other organisms [27]–[31]. Nonetheless, the information revealed by using this approach could only detect a fraction of the total RNA content, representing the tip of the iceberg. In contrast, in our study we show the clear advantage of the whole trasncriptome analysis of rRNA-depleted samples for studying Down syndrome. Hence, our approach offers the possibility to detect previously not well-characterised - or completely uncharacterized - non-coding RNA, such as snoRNAs, miRNAs and others, emerging as novel candidates for their possible contribution to the pathogenesis of different human disorders [34]–[36]. Coupling the ribodepletion procedure of samples followed by massive-scale RNA sequencing provides new intriguing opportunities to better understand the underlying molecular bases of complex phenotypes, such as herein described for Down syndrome.

In contrast, in the last years, most of studies mainly focused on hybridization- and tag-based expression profiling on *post-mortem* DS tissues and fetuses, with only few of them considering adult whole blood samples as a good source of RNA to address these aspects [37]–[39]. Since angiogenesis' suppression, endothelial dysfunction and infection recurrence are hallmarks of DS, and several studies suggest the use of endothelial progenitors - previously shown to be impaired in DS [12] - in the clinical setting [43]–[47], these cells represent an optimal source for studying blood-related DS pathological features. Therefore, the possibility to investigate in a genome-wide scale and easy-accessible non-invasive manner - early gene regulatory mechanisms responsible of cardiovascular disease, cancer and immune disorders in DS, would be of great clinical interest. Hence, our study was accurately designed to investigate these issues.

The analysis of the whole transcriptome of DS-isolated EPCs allowed us to detect differential expression - compared to the euploid sample - of even low expressed genes in immune and inflammatory pathways, crucial for DS pathogenesis, showing the great potential of RNA-Seq to detect even subtle changes in gene expression. Clearly, we are aware that we cannot conclusively attribute to trisomy 21 all the changes in transcript levels found within this single case-control study since RNA-Seq decreases the experimental noise, but cannot reduce the individual variability. In order to separate the confounding effect due to the individual variability from the effect related to DS condition, a larger number of biological replicates - for each condition - should be considered. However, in this case, most of gene expression changes identified in the present work confirmed other data derived from previous independent studies performed on the same specific cell type in more DS and euploid samples [12].

At the same time we also disclosed novel regions of active transcription falling outside annotated *loci*, with strong evidence of DE within DS progenitor cells. In addition, our work revealed a wide *spectrum* of not yet well-characterized non-coding RNAs (particularly snoRNAs) with evidence of differential expression, some of them localized on HSA21 and shown to be over-expressed in DS cells, possibly accounting for some of the observed angiogenesis- and immune-related DS phenotypes.

Moreover, our approach allowed us to identify novel DS-specific splicing isoforms for a large subset of genes, even belonging to crucial pathways involved in DS pathogenesis (i.e. *DYRK1A*) [65]. Alternative splicing is currently known to generate either novel transcripts – possibly encoding novel domains – or to have regulatory roles through balancing levels of those mRNAs encoding functional proteins [74] and, very recently, it has been highlighted the power of RNA-Seq in detecting splicing differences in brain regions of individuals affected by Alzheimer's disease [75].

In addition, low-expressed transcripts, subtle changes in the expression of both known and, more interestingly, yet unannotated transcripts, were also investigated. It should be noted that a fundamental aspect of gene expression regulation, emerging as a crucial issue for inherited disorders and cancer in humans, is the identification of *cis*- and *trans*-acting regulatory regions within 5′ and 3′ UTRs of genes. To this aim, the present study shows the great potential of RNA-seq towards the identification of novel putative extended UTRs for already known genes, possibly representing novel miRNA targets or regulatory sites for gene transcription, and to our knowledge this is the first paper describing the complete transcriptome of HSA21 trisomic endothelial progenitor cells.

On the other hand, it is clear that the high extent of complexity, not completely detected by commonly used approaches, opens several new challenges either from computational and experimental point of view, not easily solvable within a single study. For instance, the much higher level of mapping disclosed, and then measured, into unannotated TARs, requires suitable procedures to build appropriate novel gene models. Further studies will be then required to combine information from annotated genes, extended 3′ and 5′ - and exon boundaries - with those arising from igTARs and inTARs. A possible way to cope with this problem could be to build-up putative gene models and assess them by using data-driven library of junctions and iteratively repeat the mapping in a similar way as proposed by TopHat [76]. Another challenge to face is the reconstruction, and thus the further quantification, of multiple isoforms of a transcript, including those arising unannotated TARs or coming from revised gene models, in a statistical rigorous way.

Clearly, as occurs for any data-driven procedure, such approaches are likely to require very high coverage, a large number of samples and the integration with different type of biological information and data in order to be robust.

In conclusion, although with the limitation for the number of analyzed samples, we have shown the great potential of performing whole transcriptome RNA sequencing using ribosomal-depleted samples from a technical, technological and bioinformatics point of view. We believe the above-described procedures may represent a useful guideline even for larger, and more statistically significant, case-control studies based on RNA-Seq.

Since transcriptome profiling represents a powerful tool for the functional analysis of EPCs in health and disease [77], coupling this innovative technological approach, as shown herein within the context of Down syndrome, to the easy availability of circulating progenitor cells from blood samples, render this kind of analysis very feasible for large-scale studies of transcriptome in both physiological and pathological states.

## Materials and Methods

### Total RNA isolation and ribodepletion

Cells were isolated as described in [7] from peripheral blood samples of DS and euploid donors recruited at the Second University of Naples, and an approval statement was obtained by the ethics' review board of the “Monaldi Hospital”, Second University of Naples. Written informed consent was obtained from individuals involved in this study according to the principles expressed in the Declaration of Helsinki.

Briefly, total mononuclear cells were isolated by density gradient centrifugation of peripheral blood samples on Histopaque-1077 (Sigma). Cells were washed twice with PBS, plated on culture dishes pre-coated with gelatin and fibronectin and maintained in endothelial growth medium-2 (EGM2; Cell Systems). Cells were cultured at 37°C with 5% CO2 in a humidified atmosphere. After four days, non-adherent cells were removed and adherent cells were collected for RNA isolation.

Total RNA was isolated from endothelial progenitor cells as described [7]. Integrity and quantity of RNA was evaluated by Experion (Biorad), following the manufacturer's instructions. Ribosomal RNA depletion was performed on 10 µg of isolated total RNA by using magnetic beads (RiboMinus™ Eukaryote Kit for RNA-Seq, Invitrogen) according to the manufacturer's protocol (see for technical details File S1). 10 µg of total RNA were incubated at 72°C for 5 min to allow a complete denaturation for efficient hybridization to single-stranded eukaryote rRNA sequence-specific 5′-biotin labeled oligonucleotide probes (targeted against 5S, 5.8S, 18S and 28S human rRNAs) containing locked nucleic acids (LNA) at specific positions. Then, streptavidin-coated RiboMinus™ Magnetic Beads were used to capture rRNA-probes complexes to be further discarded form total RNA samples. The efficiency of rRNA depletion was evaluated on the Experion. Resulting RNA was successfully fragmented with RNase III and, after cleanup with RiboMinus™ Concentration Module (Invitrogen) according to the manufacturer's protocol, resulting fragmented samples were quantified on the Qubit Fluorometer (Invitrogen). The appropriate size distribution of fragmented RNA was finally evaluated on the Experion. The experimental procedure used in this work is illustrated in Figure S1.

### Stand-oriented cDNA library preparation

100 ng of the fragmented RNA samples were hybridized and ligated to double stranded oligonucleotides adapter suited for the 5′ SOLiD System sequencing (details in File S1). Reverse transcription was performed using ArrayScript™ Reverse Transcriptase. Purified cDNA samples were denatured on 6% TBE-Urea gel, and size selection (150–250 bp) was performed. PCR amplification on gel slices was then performed using AmpliTaq® DNA Polymerase, and yield of purified PCR products was assessed on the Qubit Fluorometer and NanoDrop spectrophotometer (Invitrogen). Size distribution of cDNA libraries was evaluated on the Experion.

### SOLiD sequencing

We drove 500 pg of each library onto 1-µm-diameter beads using emulsion PCR, according to the SOLiD™ Whole Transcriptome Analysis Kit (Applied Biosystems). Libraries were sequenced using the Applied Biosystems SOLiD sequencing, as 50-mers. We sequenced ∼200,000,000 (100 M for each sample, euploid and DS) beads using ‘sequencing by ligation’ chemistry on a SOLiD sequencer version 3 (Applied Biosystems). Approximately 97% of beads deposited onto the slice generated good-quality sequence reads 50 nt in length (Figure S2 and Table S1).

SOLiD processed files have been submitted to the Gene Expression Omnibus (GEO; http://www.ncbi.nlm.nih.gov/geo/) repository (accession n. GSE27443).

### Quantitative Real-Time for RNA-Seq validation

Quantitive Real-Time PCRs were performed on the same euploid and DS rRNA-depleted samples that underwent library construction and further sequencing on the SOLiD platform. Amplification reaction mix contained 1× SYBR Green PCR master mix (Applied Biosystems), 160 nM of each primer and about 50 ng of cDNA (RNA equivalent) as template. PCR conditions were 95°C for 10 min followed by 40 cycles of 95°C 30 sec, 60°C 30 sec and 72°C 30 sec. Melting curves were generated after amplification. Data were collected using the 7900HT Fast real time PCR system (Applied Biosystems); each assay for each of the 24 analysed genes (Figure S7) was performed in duplicate in both rRNA-depleted samples. Primers were designed using Oligo 4.0-s. The relative gene expression was calculated using the 2^−ΔΔCt^ method [78].

### Mapping strategy and data visualization

The whole mapping strategy is illustrated in Figure S2 and consists in several steps. First, the total reads produced were filtered out accordingly to quality values, secondly, those reads that mapped to the adapters and to the ribosomal sequences were further removed, thirdly RNA-MATE software [59] version 1.1 was used to map the usable reads either to the genome and to a custom-designed library of exon-junction sequences, (see File S1).

RNA-MATE is an open source software specifically designed to map RNA-Seq data generated from the SOLiD system. It works cyclically. At each cycle it attempts to map usable reads first to the reference genome and subsequently to the junctions' library. At the end of each cycle, reads failed to map to the genome or to the junctions library were left-end trimmed using a pre-defined lengths schema.

RNA-MATE allows a user to control the number of mismatches tolerated for each cycle, however it does not incorporate the possibility of mapping gaps, reducing the possibility of locating reads with small indel. Moreover, it requires the pre-construction of a junctions library limiting the possibility of identifying de-novo junctions. However, the assessment and the correct interpretation of mapping strategies that are junctions model free has not been completely elucidated and good performance are obtained only at the price of a much higher coverage. Moreover, tail-end trimming the reads at each cycle allows either to cope with the behaviour of the quality values (that are usually worst in last bases of the reads) and to partially handle the presence of novel splicing junctions allowing to map the right side of the read.

By default, RNA-MATE allows to directly assign multiple reads with a single “best hit” to that specific position. In our pipeline, all remaining multiple reads (with at most 10 mapping positions) underwent the rescue procedure with default parameters.

At the end of the alignment procedure three types of reads were identified: UARs, MRs and unmapped reads (see File S1 for definitions).

### Annotation and quantification of RefSeq transcriptional events

Given the results of the alignment, first we performed a within sample analysis aimed to extract and characterize the activity of both states independently, then we provided a cross-comparison between trisomic and euploid cells aimed to detect differences in term of gene expression.

In order to provide a quantitative estimate of gene expressions in both trisomic and euploid cells, we considered genes in the ReqSeq annotation. However we suitably revised the annotation to remove ambiguities due to overlapping genes, see File S1 for technical details. The annotation contains 215952 annotated elements (i.e., exons or part of them) in a BED format corresponding to 21122 uniquely identified (and non redundant) RefSeq genes or group/family of RefSeq genes.

For each gene in the RefSeq annotation a preliminary estimate of the global expression was obtained by computing the number of UARs starting in all the annotated elements (i.e., exons or part of exons) corresponding to the same gene. Then, the final expression value was corrected by adding to each specific *locus* the read counts derived from the splice junctions. Additionally, an exon by exon usage map and the corresponding reads counts was provided in order to facilitate isoforms identification.

To account for transcripts of different lengths when selecting active genes, the gene expression counts values of annotated loci were converted in RPKM [55]. For each sample, only loci with RMKM>0.1 were considered detected.

Expressed genes in both samples were further classified according to RPKM distributions in 5 categories: 1) very low expression, 2) low expression, 3) intermediate, 4) high and 5) very high expression (Figure 2 and details in File S1).

The analysis of RefSeq loci was also aimed to detect a particular enrichment in 3′ (or 5′) UTRs (see File S1).

### Identification of alternative splicing events

We inferred the evidence of multiple isoforms within each annotated gene on the basis of the reads that mapped to the splicing junctions and we suggested the presence of novel isoforms from the type of junction mapped (i.e., junctions annotated in some database such that RefSeq, UCSC or Ensembl or novel combinatorial junctions). In particular, we considered as alternative splicing marks either the multiple donors or the multiple acceptor (or both) junctions (see File S1 for definition).

For the sake of simplicity to reduce the effect of the random matching, a junction was considered reliable if there were at least T1 reads mapped on it. Then the identification proceeded as follows. First, for each sample, we retrieved all the reliable junctions and, among them we selected those containing either multiple donors or multiple acceptors. Then, RefSeq genes containing such junctions were detected. Such genes constitute an initial list of candidates to the presence of multiple splicing isoforms. The lists can be further filtered using information arising from exon by exon map usage to remove mapping artefacts. Secondly, the two samples were cross-compared as follows: the spliced junctions common to both samples were identified then, for each sample, a list of candidate sample specific junctions was obtained. To remove the effect of the user specific threshold T1, each list was subsequently filtered, by removing those junctions that received any number of hits in the other sample.

Finally, since each junction was also classified as RefSeq junction, UCSC, Ensembl junction or as putative new junction accordingly to if it was annotated in the corresponding database or it was a results of a pure combinatorial process, we use such information to detect those genes containing putative new junctions that are candidate to show novel (unannotated) isoforms.

### Refinement of non-RefSeq *loci*

Given the RefSeq annotation we defined and annotated on each strand on the genome igRs and inRs (File S1) to cover all the genome. The annotation was performed independently on each strand and regions were labelled, enumerated and described in a BED file. The regions were quantified in each sample to provide a measure of the overall mapping in non RefSeq regions. In order to quantify the strength of the signal in the truly unannotated regions, both igR and inR were filtered on the basis of the UCSC and Ensembl Annotation (see File S1).

Remaining regions were re-labelled and enumerated. The reads count was repeated on both samples. For comparative purposes and to assess the consistency of UCSC and Ensembl databases, the reads count was also performed on the UCSC and Ensembl Annotation filtered by the RefSeq annotation.

Subsequently, to more precisely determine novel active regions, each unannotated genomic region (either igR and inR) that showed presence of signal (i.e., mapped reads) underwent an ad-hoc refinement procedure. The refinement procedure is aimed to more precisely define the approximate location of the active regions within the unannotated regions (i.e., to identify igTARs and inTARs, where there is a concentration of reads, removing those regions or part of regions which showed sparse or no signal at all).

The refinement procedure was performed either on each samples independently - to determine sample specific annotations (data not shown) - and by pooling together the two samples in order to determine a set of unannotated active regions, igTARs and inTARs, on which trisomic and euploid cells can be compared. The reads count was finally repeated for each sample.

### Statistical tests for differential expression

In order to detect DE between trisomic and euploid states, we first compared the two samples at RefSeq level, then we compared the previously identified un-annotated intergenics and intronic regions.

Statistical significance has been inferred from the total observed reads count in each locus combining together a bunch of tests, namely DEGseq [79], DESeq [80] and edgeR [81] for which R-packages are available under Bioconductor (www.bioconductor.org/). Such tests are based on slightly different assumptions that usually produce a different level of stringency - and sometime different results - when applied to small sample experiments. However, all of them are particularly suited for RNA-Seq data, hence they were independently applied to the dataset. For each locus we compute a p-value and its corresponding adjusted p-value or q-value to detect significant change in the expression (i.e., DE loci).

A cut-off of 0.1 was used for DESeq (that was found very conservative for small sample), while a cut off of 0.0001 was used for both edgeR and DEGseq (both of them resulted to be more permissive. Additionally, a threshold of 1.5 on the fold change between the normalized samples was imposed to filter out those genes whose significance appeared marginal (see File S1 for details).

Finally, the results of each selection were cross-compared either to compromise with their assumptions and to illustrate their impact in the final choice.

DE evidence was finally classified as “strong”, “good”, and “acceptable”. All DE genes below the fold-change threshold, but found significant in at least one test, were classified as “weak” evidence. Figure 6A shows the scattered plot of the normalized log intensities vs the normalized log ratio between the two samples for RefSeq loci.

## Supporting Information

Figure S1 Experimental procedure. Schematic representation of the whole RNA-Seq experiment. Depicted are: Total RNA isolation (1) and ribo-depletion (2). Ribo-depleted total RNA is fragmented (3), then ligated to specific adaptors (4) and retro-transcribed (5). The resulting cDNA is size selected by gel electrophoresis (6), and cDNAs are PCR amplified (7). Then size distribution is evaluated on Experion (8). Emulsion PCR is finally used for the clonal amplification of SOLs (9). Enriched beads are deposited onto glass slides (10), and sequenced by ligation on the SOLiD v3 platform.
(JPG)

Figure S2 Data analysis pipeline. Schematic representation of the data analysis workflow described in detail in “Materials and Methods”.
(JPG)

Figure S3 Summary of mapping results. Distribution of the sequenced reads according to the mapping procedure. DS sample (A) and Euploid (B).
(JPG)

Figure S4 Distribution of the UARs in the human genome. Distribution of the UARs according to RefSeq genes, intronic intergenic regions and mitochondrial chromosome. DS sample (A) and Euploid (B).
(JPG)

Figure S5 Detection of alternative splicing events. Schematic representation of the computational analysis used to detect sample-specific ASEs both canonical and unannotated. Reliability of the junction was measured with T1 = 3 (A) and with T1 = 5 (B).
(JPG)

Figure S6 Differential expression of lincRNAs. Standard MA-plot of the normalized global observed counts per each lincRNA.
(JPG)

Figure S7 Quantitative Real-Time PCR validation. A random selection of “no change” (A) and weak DE (B) RefSeq genes between the analyzed samples confirmed by qRT-PCR. Relative expression levels for a selection of DE RefSeq genes in DS state (C).
(JPG)

Figure S8 Differential expression of igTARs and inTARs. Standard MA-plot of the normalized global observed counts per each identified igTAR (A) and inTAR (B). Venn diagrams showing the number of regions with evidence of DE according to each statistical method used (igTARs in panel C and inTARs in panel D).
(JPG)

Table S1 Mapping summary.
(DOC)

Table S2 Summary of mapping on the junctions.
(DOC)

Table S3 Distribution of RPKM expression level of snoRNA host genes.
(DOC)

Table S4 List of differentially expressed snoRNAs in human trisomy 21.
(DOC)

Table S5 Primer pairs used for quantitative RT-PCR.
(DOC)

File S1 Supporting Materials and Methods.
(DOC)
