# Supplementary figures and images for: Massive-Scale RNA-Seq Analysis of Non Ribosomal Transcriptome in Human Trisomy 21
Source: PLoS One. 2011 Apr 20;6(4):e18493. doi: 10.1371/journal.pone.0018493 (PMC3080369; doi:10.1371/journal.pone.0018493)

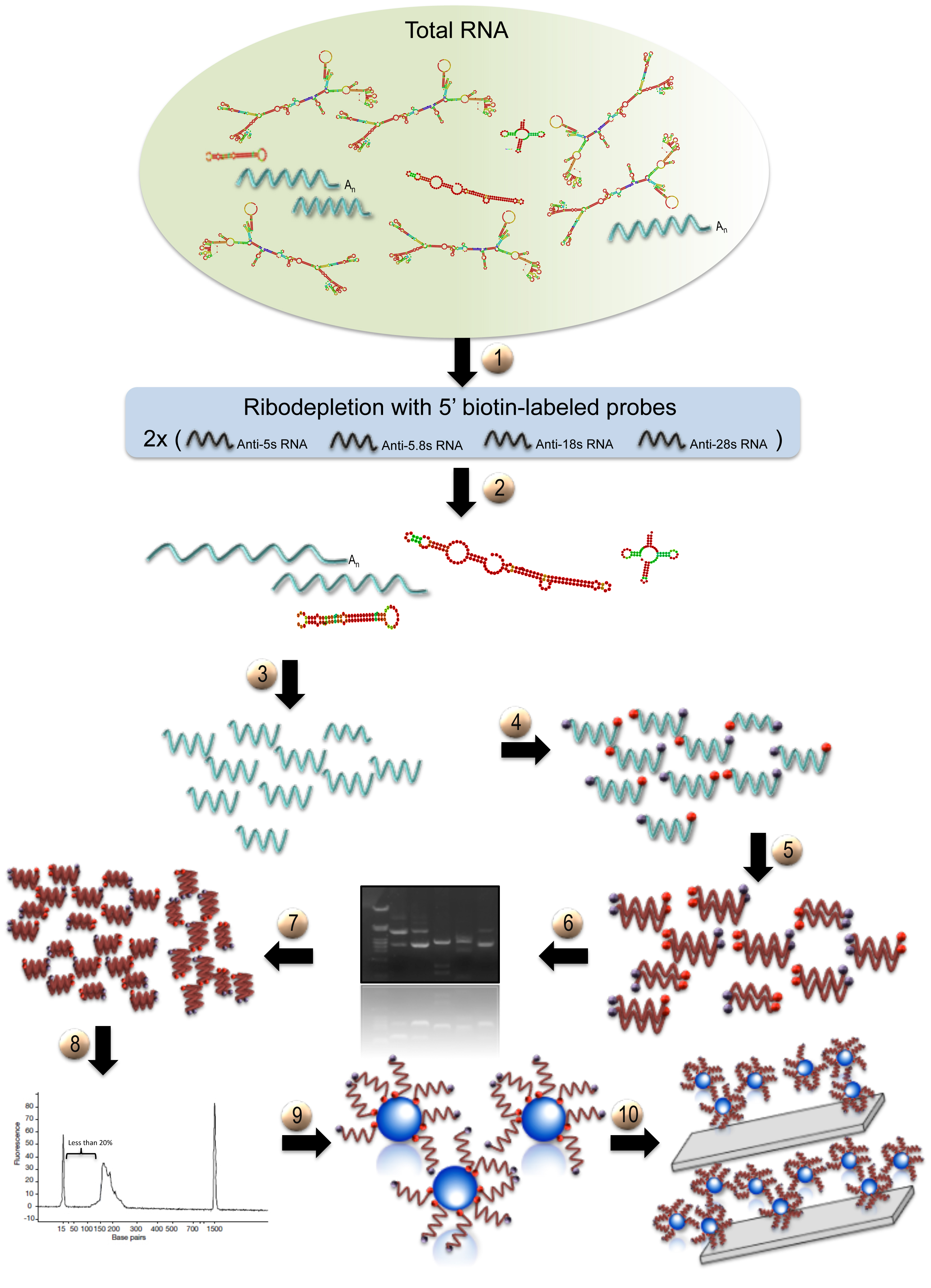

Supplement: Figure S1 — Experimental procedure. Schematic representation of the whole RNA-Seq experiment. Depicted are: Total RNA isolation (1) and ribo-depletion (2). Ribo-depleted total RNA is fragmented (3), then ligated to specific adaptors (4) and retro-transcribed (5). The resulting cDNA is size selected by gel electrophoresis (6), and cDNAs are PCR amplified (7). Then size distribution is evaluated on Experion (8). Emulsion PCR is finally used for the clonal amplification of SOLs (9). Enriched beads are deposited onto glass slides (10), and sequenced by ligation on the SOLiD v3 platform. (JPG) [file pone.0018493.s001.jpg]

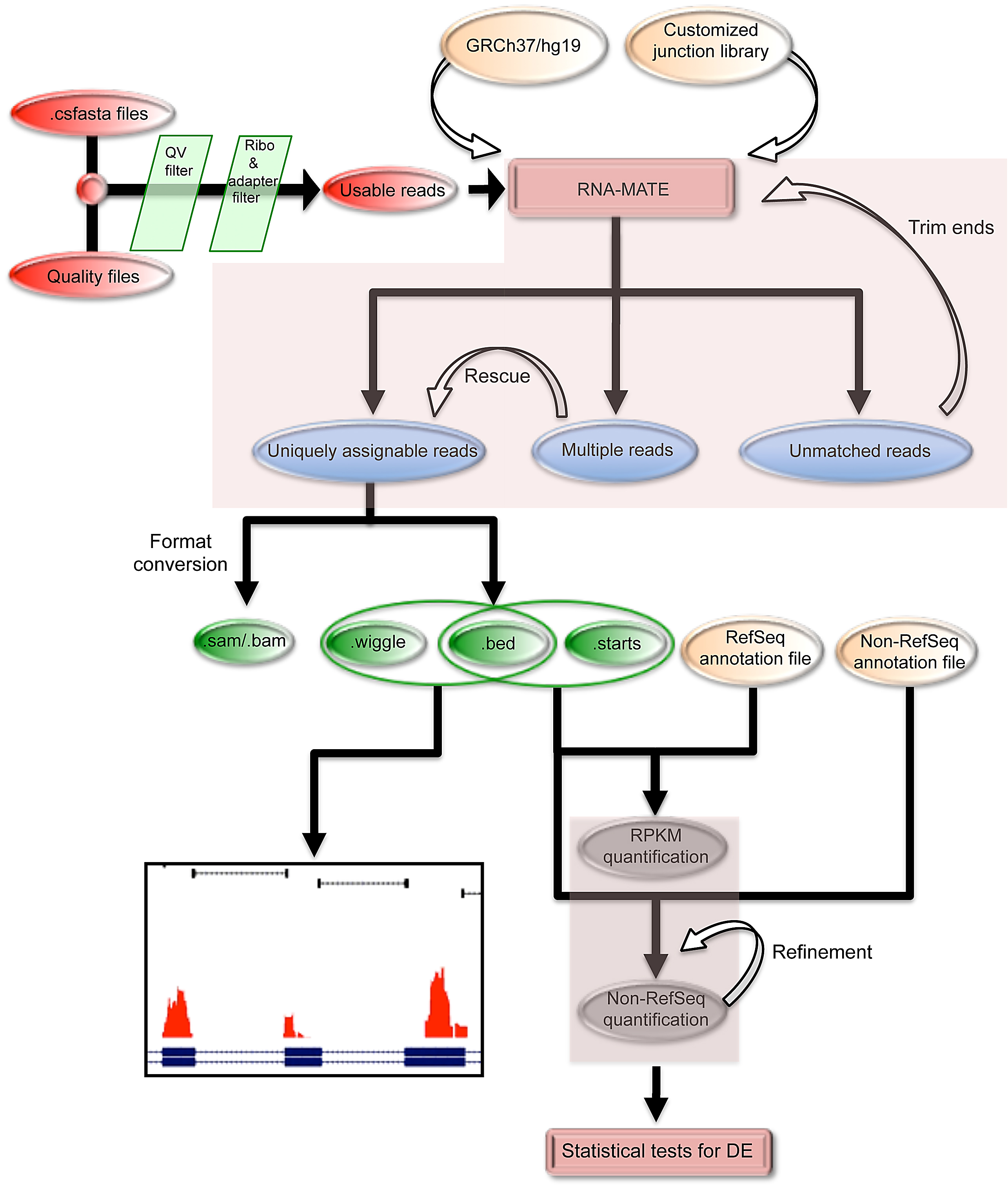

Supplement: Figure S2 — Data analysis pipeline. Schematic representation of the data analysis workflow described in detail in “Materials and Methods”. (JPG) [file pone.0018493.s002.jpg]

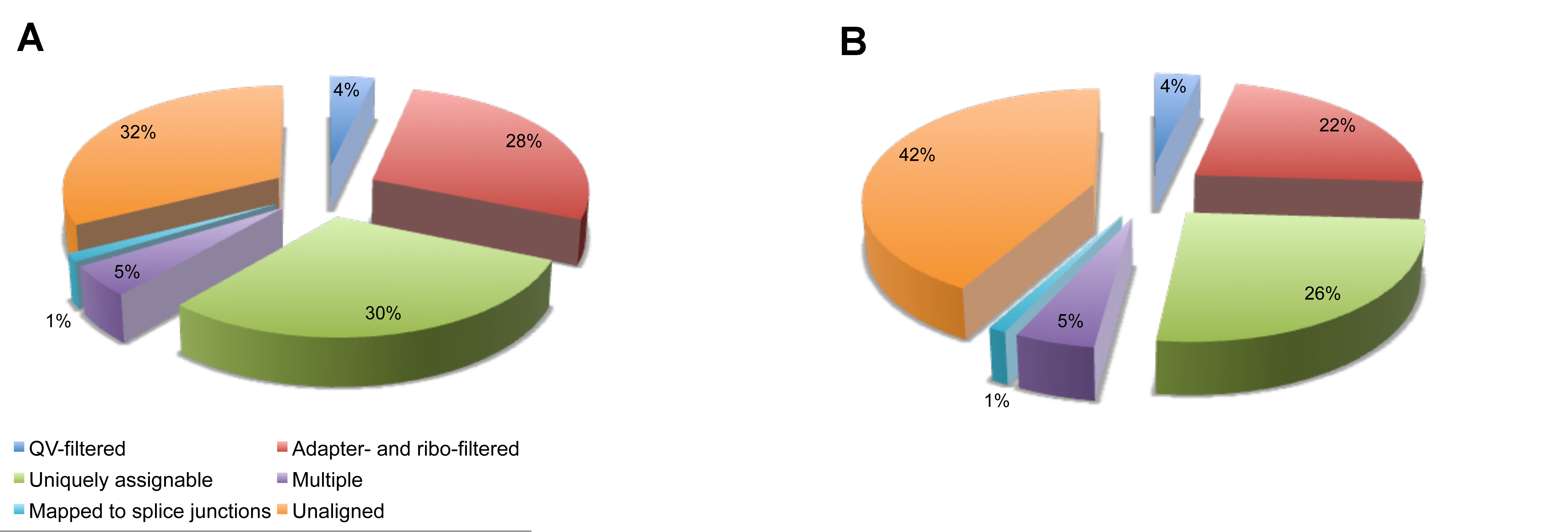

Supplement: Figure S3 — Summary of mapping results. Distribution of the sequenced reads according to the mapping procedure. DS sample (A) and Euploid (B). (JPG) [file pone.0018493.s003.jpg]

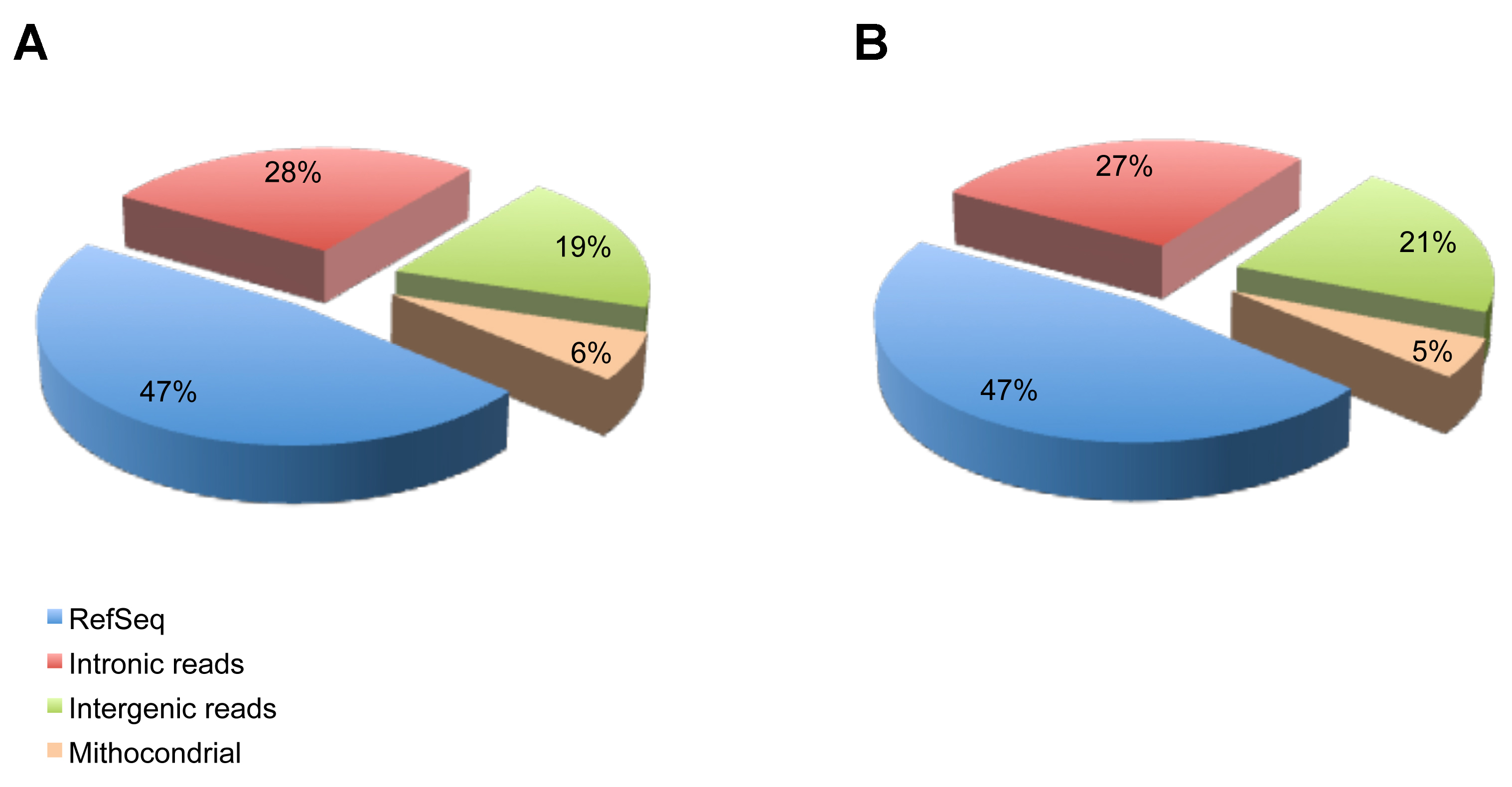

Supplement: Figure S4 — Distribution of the UARs in the human genome. Distribution of the UARs according to RefSeq genes, intronic intergenic regions and mitochondrial chromosome. DS sample (A) and Euploid (B). (JPG) [file pone.0018493.s004.jpg]

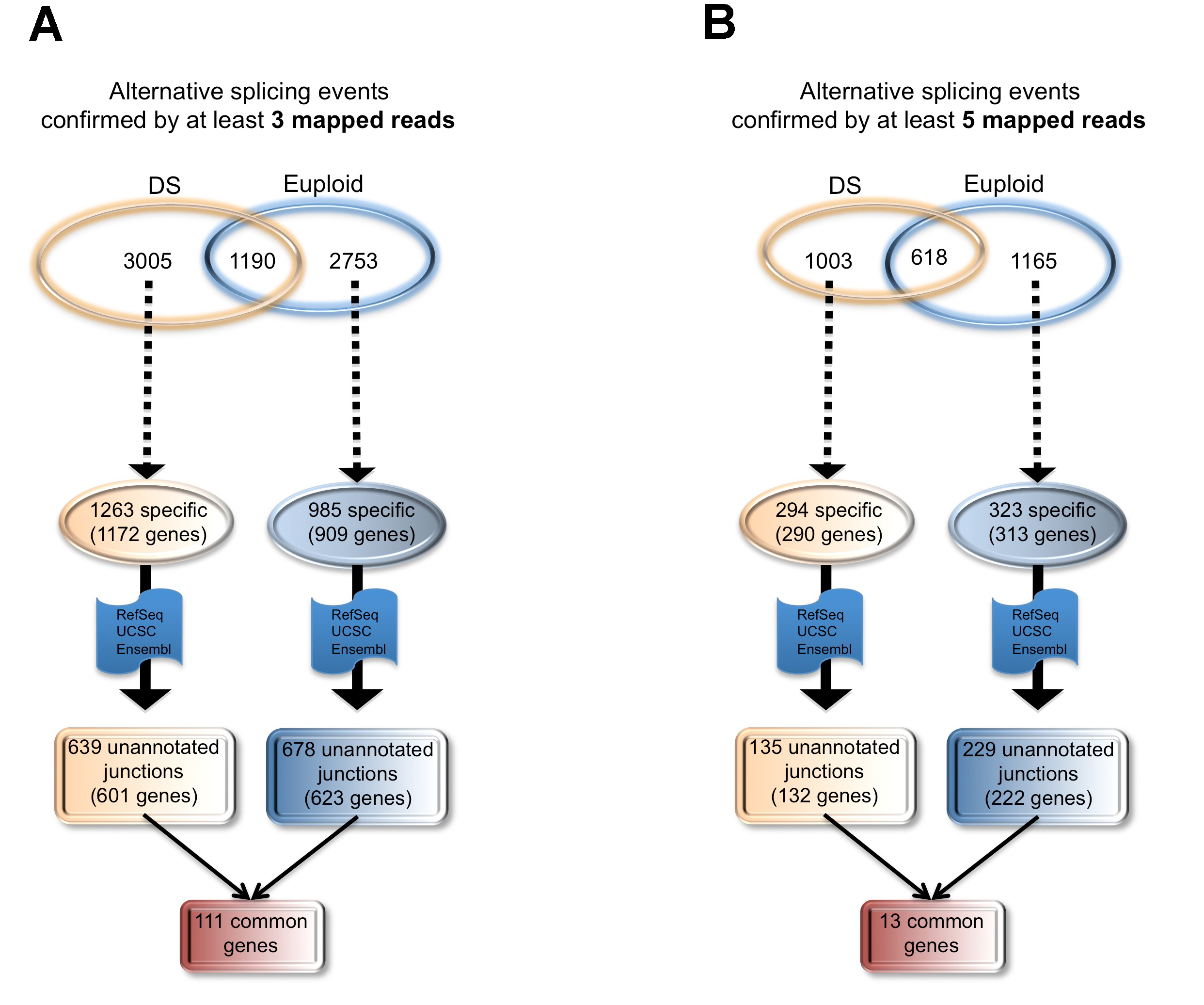

Supplement: Figure S5 — Detection of alternative splicing events. Schematic representation of the computational analysis used to detect sample-specific ASEs both canonical and unannotated. Reliability of the junction was measured with T1 = 3 (A) and with T1 = 5 (B). (JPG) [file pone.0018493.s005.jpg]

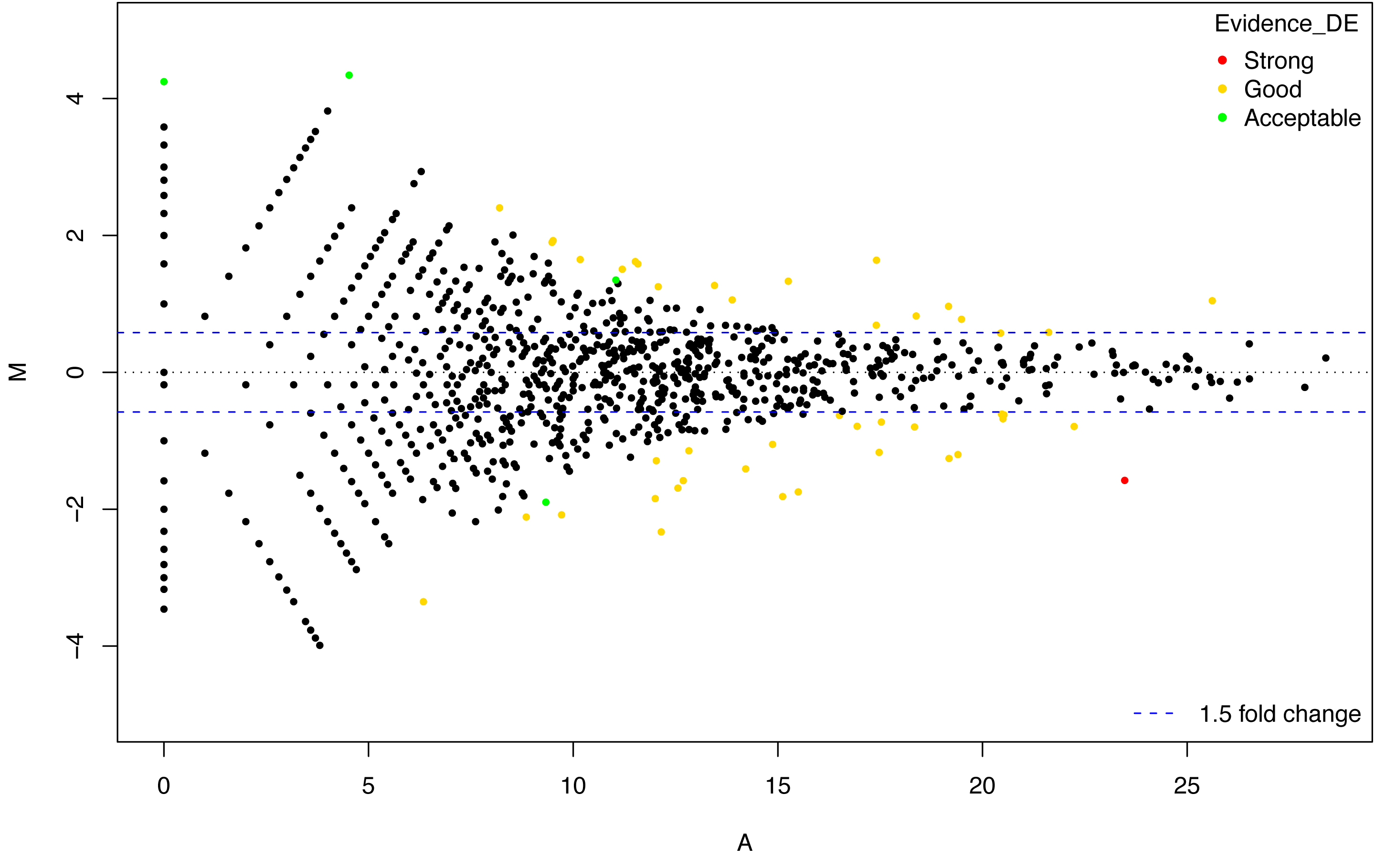

Supplement: Figure S6 — Differential expression of lincRNAs. Standard MA-plot of the normalized global observed counts per each lincRNA. (JPG) [file pone.0018493.s006.jpg]

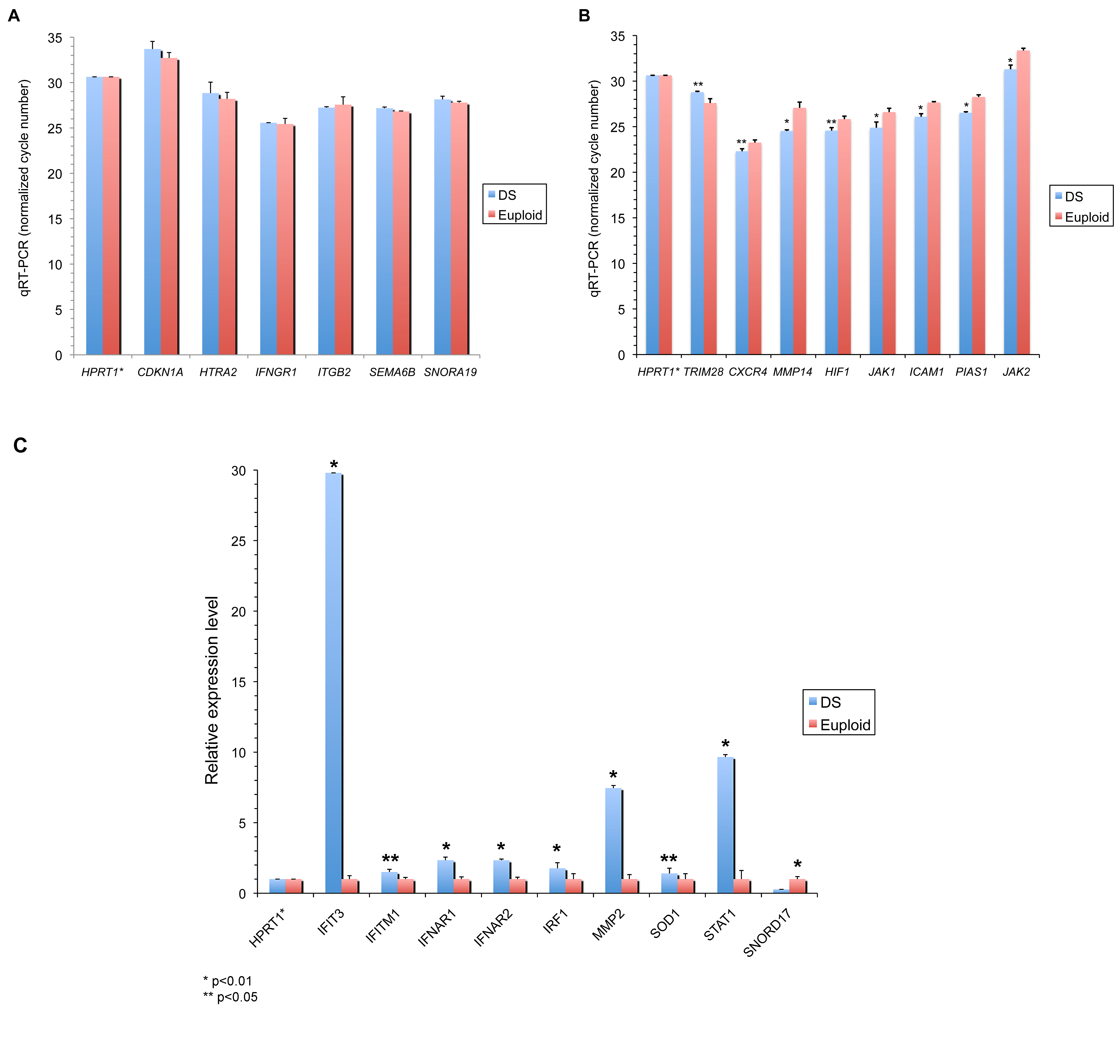

Supplement: Figure S7 — Quantitative Real-Time PCR validation. A random selection of “no change” (A) and weak DE (B) RefSeq genes between the analyzed samples confirmed by qRT-PCR. Relative expression levels for a selection of DE RefSeq genes in DS state (C). (JPG) [file pone.0018493.s007.jpg]

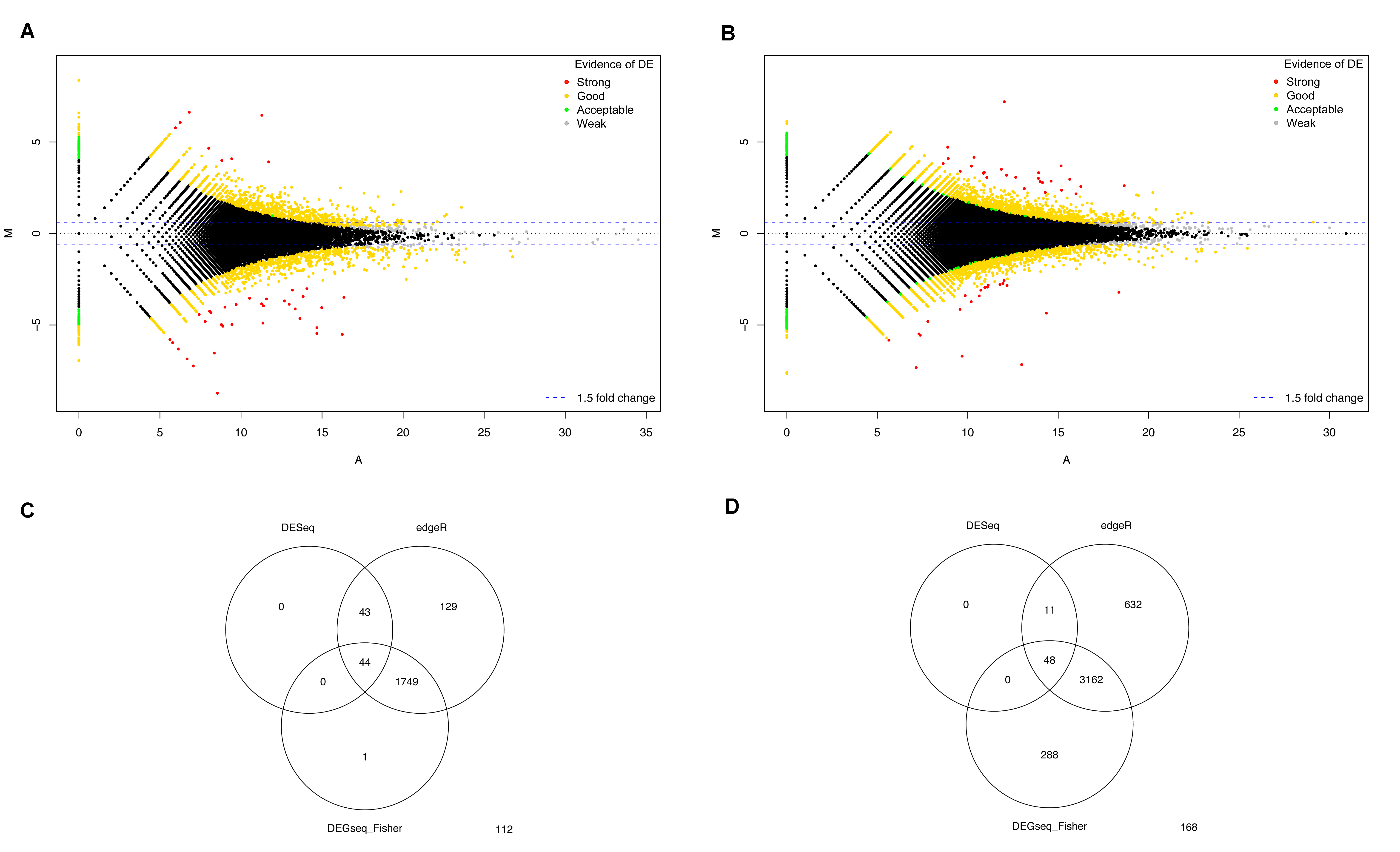

Supplement: Figure S8 — Differential expression of igTARs and inTARs. Standard MA-plot of the normalized global observed counts per each identified igTAR (A) and inTAR (B). Venn diagrams showing the number of regions with evidence of DE according to each statistical method used (igTARs in panel C and inTARs in panel D). (JPG) [file pone.0018493.s008.jpg]
