# Supplementary material for: Massive-Scale RNA-Seq Analysis of Non Ribosomal Transcriptome in Human Trisomy 21
Source: PLoS One. 2011 Apr 20;6(4):e18493. doi: 10.1371/journal.pone.0018493 (PMC3080369; doi:10.1371/journal.pone.0018493)
Supplement: Table S1 — Mapping summary. (DOC) [file pone.0018493.s009.doc]

Table S1

|  | **Euploid** | **DS** |
| --- | --- | --- |
| **Total Reads** | 98196489 | 98174677 |
| **QV filtered*** | 3587870 | 3611051 |
| **Adapter-filtered**** | 173617 | 117595 |
| **Ribo-filtered**** | 21749962 | 26945958 |
| **Usable reads** | 72685040 | 67500073 |
| **Reads mapped to hg19** | 30441547 | 34426919 |
| **Unique reads mapped to hg19** | 21072159 | 24429402 |
| **Uniquely assignable reads (after rescue)** | 25662885 | 29673445 |
| **Reads mapped to splice junctions***** | 1087343 | 1259987 |
| **Unaligned Reads** | 41156150 | 31813167 |

^*^Reads filtered with a median QV <=5;

^**^Number of usable beads for the following “Reads mapping analysis”;

^***^The junction library contained 2,061,961 junctions (obtained by a combinatorial assemble of the RefSeq transcripts);
