# Supplementary material for: Massive-Scale RNA-Seq Analysis of Non Ribosomal Transcriptome in Human Trisomy 21
Source: PLoS One. 2011 Apr 20;6(4):e18493. doi: 10.1371/journal.pone.0018493 (PMC3080369; doi:10.1371/journal.pone.0018493)
Supplement: Table S2 — Summary of mapping on the junctions. (DOC) [file pone.0018493.s010.doc]

**Table S2**

|  | **Euploid** | **DS** |
| --- | --- | --- |
| Total mapped splice junctions | 80200 | 92939 |
| Mapped junctions^1^ | 64115 | 56621 |
| Mapped junctions^2^ | 43308 | 48604 |
| Total detected ASEs^1^ | 3943 | 4195 |
| Total detected ASEs^2^ | 1783 | 1621 |
| Non-RefSeq ASEs^1^ | 1129 | 1104 |
| Non-RefSeq ASEs^2^ | 458 | 340 |
| Genes with non-RefSeq ASEs^1^ | 771 | 765 |
| Genes with non-RefSeq ASEs^2^ | 320 | 223 |
| Novel^#^ ASEs^1^ | 909 | 848 |
| Novel^#^ ASEs^2^ | 353 | 238 |
| Genes with novel ASEs^1^ | 657 | 620 |
| Genes with novel ASEs^2^ | 264 | 168 |

ASEs (Alternative Splicing Events);

^1^ with at least 3 mapped reads; ^2^with at least 5 mapped reads.

^#^Combinatorial splice junctions not annotated in RefSeq, UCSC and Ensembl databases.
