# Supplementary material for: Massive-Scale RNA-Seq Analysis of Non Ribosomal Transcriptome in Human Trisomy 21
Source: PLoS One. 2011 Apr 20;6(4):e18493. doi: 10.1371/journal.pone.0018493 (PMC3080369; doi:10.1371/journal.pone.0018493)
Supplement: Table S3 — Distribution of RPKM expression level of snoRNA host genes. (DOC) [file pone.0018493.s011.doc]

**Table S3**

Euploid

|  | **Very high snoRNAs** | | **High snoRNAs** | |
| --- | --- | --- | --- | --- |
| ***RPKM Category*** | *Total host* | *% host* | *Total host* | *% host* |
| Very high | 2 | 8 | 0 | 0 |
| High | 17 | 4,75 | 5 | 1,4 |
| Intermediate | 55 | 1,04 | 28 | 0,53 |
| Low | 10 | 0,21 | 15 | 0,32 |
| Very low | 1 | 0,04 | 1 | 0,04 |
| Not detected | 0 | 0 | 0 | 0 |

DS

|  | **Very high snoRNAs** | | **High snoRNAs** | |
| --- | --- | --- | --- | --- |
| ***RPKM Category*** | *Total host* | *% host* | *Total host* | *% host* |
| Very high | 2 | 8,33 | 0 | 0 |
| High | 16 | 4,08 | 7 | 1,78 |
| Intermediate | 54 | 1 | 35 | 0,65 |
| Low | 10 | 0,21 | 14 | 0,3 |
| Very low | 0 | 0 | 0 | 0 |
| Not detected | 0 | 0 | 0 | 0 |
