# Supplementary material for: Massive-Scale RNA-Seq Analysis of Non Ribosomal Transcriptome in Human Trisomy 21
Source: PLoS One. 2011 Apr 20;6(4):e18493. doi: 10.1371/journal.pone.0018493 (PMC3080369; doi:10.1371/journal.pone.0018493)
Supplement: Table S4 — List of differentially expressed snoRNAs in human trisomy 21. (DOC) [file pone.0018493.s012.doc]

Table S4. List of differentially expressed snoRNAs in human trisomy 21

| ***Gene symbol*** | ***Accession number*** | ***Evidence DE*** | ***Gene symbol*** | | ***Accession number*** | ***Evidence DE*** |
| --- | --- | --- | --- | --- | --- | --- |
| SCARNA1 | NR_002997 | DOWN | SNORD104 | NR_004380 | | DOWN |
| SCARNA10 | NR_004387 | DOWN | SNORD109 | NR_001295 | | DOWN |
| SCARNA11 | NR_003012 | DOWN | SNORD116-3 | NR_003318 | | DOWN |
| SCARNA14 | NR_004388 | DOWN | SNORD116-11 | NR_003326 | | DOWN |
| SCARNA16 | NR_003013 | DOWN | SNORD116-15 | NR_003330 | | DOWN |
| SCARNA18 | NR_003139 | UP | SNORD116-2 | NR_003317 | | DOWN |
| SCARNA20 | NR_002999 | UP | SNORD116-20 | NR_003334 | | DOWN |
| SCARNA4 | NR_003005 | DOWN | SNORD116-25 | NR_003339 | | DOWN |
| SCARNA8 | NR_003009 | DOWN | SNORD116-8 | NR_003323 | | DOWN |
| SNORA11 | NR_002953 | DOWN | SNORD15A | NR_000005 | | DOWN |
| SNORA11D | NR_003711 | DOWN | SNORD17 | NR_003045 | | DOWN |
| SNORA11D | NR_003711 | DOWN | SNORD2 | NR_002587 | | DOWN |
| SNORA20 | NR_002960 | UP | SNORD22 | NR_000008 | | DOWN |
| SNORA21 | NR_002576 | DOWN | SNORD23 | NR_003048 | | DOWN |
| SNORA22 | NR_002961 | UP | SNORD30 | NR_002561 | | DOWN |
| SNORA24 | NR_002963 | UP | SNORD31 | NR_002560 | | DOWN |
| SNORA26 | NR_003016 | DOWN | SNORD35B | NR_001285 | | DOWN |
| SNORA27 | NR_002575 | UP | SNORD36C | NR_000016 | | DOWN |
| SNORA28 | NR_002964 | UP | SNORD38A | NR_001456 | | DOWN |
| SNORA32 | NR_003032 | DOWN | SNORD38B | NR_001457 | | DOWN |
| SNORA38B | NR_003706 | DOWN | SNORD41 | NR_002751 | | DOWN |
| SNORA39 | NR_002972 | DOWN | SNORD43 | NR_002439 | | DOWN |
| SNORA44 | NR_002976 | DOWN | SNORD46 | NR_000024 | | DOWN |
| SNORA45 | NR_002977 | DOWN | SNORD48 | NR_002745 | | DOWN |
| SNORA46 | NR_002978 | DOWN | SNORD49B | NR_003043 | | DOWN |
| SNORA52 | NR_002585 | DOWN | SNORD4B | NR_000009 | | DOWN |
| SNORA53 | NR_003015 | DOWN | SNORD51 | NR_002589 | | DOWN |
| SNORA55 | NR_002983 | DOWN | SNORD52 | NR_002742 | | DOWN |
| SNORA58 | NR_002985 | DOWN | SNORD55 | NR_000015 | | DOWN |
| SNORA66 | NR_002444 | DOWN | SNORD58C | NR_003701 | | DOWN |
| SNORA70 | NR_000011 | UP | SNORD69 | NR_003057 | | DOWN |
| SNORA71A | NR_002911 | DOWN | SNORD7 | NR_003037 | | DOWN |
| SNORA74A | NR_002915 | DOWN | SNORD75 | NR_003941 | | DOWN |
| SNORA74B | NR_002988 | DOWN | SNORD80 | NR_003940 | | UP |
| SNORA75 | NR_002921 | DOWN | SNORD82 | NR_004398 | | DOWN |
| SNORA79 | NR_003021 | DOWN | SNORD83A | NR_000027 | | DOWN |
| SNORA8 | NR_002920 | UP | SNORD88C | NR_003069 | | DOWN |
| SNORA80 | NR_002996 | UP | SNORD89 | NR_003070 | | UP |
| SNORA81 | NR_002989 | DOWN | SNORD90 | NR_003071 | | DOWN |
| SNORA84 | NR_003704 | UP | SNORD93 | NR_003075 | | DOWN |
| SNORD10 | NR_002604 | UP | SNORD96A | NR_002592 | | DOWN |
| SNORD100 | NR_002435 | DOWN | SNORD98 | NR_003076 | | DOWN |
| SNORD102 | NR_002574 | DOWN | SNORD99 | NR_003077 | | DOWN |
