# Supplementary material for: Massive-Scale RNA-Seq Analysis of Non Ribosomal Transcriptome in Human Trisomy 21
Source: PLoS One. 2011 Apr 20;6(4):e18493. doi: 10.1371/journal.pone.0018493 (PMC3080369; doi:10.1371/journal.pone.0018493)
Supplement: Table S5 — Primer pairs used for quantitative RT-PCR. (DOC) [file pone.0018493.s013.doc]

**Table S5. Primer pairs used for quantitative and semi-quantitative RT-PCR**

| **Gene symbol** | **Primer sequence (5’-3’)** | |
| --- | --- | --- |
|  | ***Forward*** | ***Reverse*** |
| *HPRT1* | TGCTGACCTGCTGGATTACA | CCTGACCAAGGAAAGCAAAG |
| *CDKN1A* | CATGACAGATTTCTACCACTCCAAA | TCCTGTGGGCGGATTAGG |
| HTRA2 | CTCTGGAGGTCCCCTGGTTAA | CACTGATTCCGGAGGAGGAA |
| IFNGR1 | TTGCTGTATGCCGAGATGGA | CGCTAACTGGCACTGAATCT |
| ITGB2 | TGAAACCCAGGAAGACCACA | AAGGACCCGAAGCCAATGC |
| *SEMA6B* | TTCTCTGACGGGATGCTCTT | ACCGCATGGACAAAGTAAGG |
| *TRIM28* | CTACCGACTCCACCACCTTCTC | AGCACAGCAGAGAACTTGGT |
| CXCR4 | CAGATAACTACACCGAGGAAA | AGCGTGATGACAAAGAGGAG |
| MMP14 | TCAAGGAGCGCTGGTTCTG | AGGGACGCCTCATCAAACAC |
| *HIF1* | TGAGGAAATGAGAGAAATGCT | TGAGGAAATGAGAGAAATGCT |
| JAK1 | CCTCTTTGCCCTGTATGACG | CATACTGTCCCTGAGCAAAC |
| *JAK2* | CTCCTCTTCTTGATGCATTTG | TGTACCTTATTCGCTTCCTTG |
| *ICAM1* | AAGTTGTTGGGCATAGAGACC | GCAGCGTAGGGTAAGGTTCT |
| PIAS1 | TCGCCATTACTCCCTGTTTC | ATGGAACTACTGATTTGCTGC |
| *SNORA19* | GCACATTTCATTGACCTGCTT | GAAGGAGACTGGCAGCATTA |
|  |  |  |
| *IFNAR1* | GGAACAGGAGCGATGAGTCT | CACTATTGCCTTATCTTCAGC |
| *IFNAR2* | ACCACCAGAGTTTGAGATTGT | GGAGACTTTATTACTGCTTGC |
| IRF1 | ACAAAGCAGGGGAAAAGGAG | GTCATCAGGCAGAGTGGAG |
| *STAT1* | AAAAGCAAGACTGGGAGCAC | ATTCCCCGACTGAGCCTGAT |
| SOD1 | AGTGAAGGTGTGGGGAAGC | CAATGATGCAATGGTCTCCT |
| IFIT3 | TGCAGGGAAACAGCCATCAT | ACTTCTGATTTCTGCTTGGTC |
| *IFITM1* | TGTCGTCTGGTCCCTGTTC | CCCGTTTTTCCTGTATTATC |
| MMP2 | GACAGTGGATGATGCCTTTG | CCATACTTCACACGGACCAC |
| *SNORD17* | GCACTGACCTTCTTCCAAGC | TGTGAGATGGGTCTCAGAGG |
