# Supplementary material for: Massive-Scale RNA-Seq Analysis of Non Ribosomal Transcriptome in Human Trisomy 21
Source: PLoS One. 2011 Apr 20;6(4):e18493. doi: 10.1371/journal.pone.0018493 (PMC3080369; doi:10.1371/journal.pone.0018493)
Supplement: File S1 — Supporting Materials and Methods. (DOC) [file pone.0018493.s014.doc]

**Supporting Information**

**Supporting “MaTERIALS and METHODS”**

**RNA preparation**

The present work focused on the whole transcriptome analysis on a high-quality ribo-depleted total RNA from primary cultured circulating endothelial progenitors. It briefly consisted in:

**A)** Total RNA isolation form EPCs;

**B)** Ribo-depletion;

**C)** Fragmentation of RNA.

**A)** Total RNA isolation form EPCs. We isolated total RNA by using standard phenol/chlorophorm extraction by Trizol (Invitrogen) following the manufacturer’s protocol. More in detail:

1) Cultured (adherent) primary cells as previously described [12,58] were lysed by directly adding Trizol (1 ml per 10 cm^2^ area for about 5*10^6^ cells/ml) to the culture flask.

2) After adding Trizol, cells were incubated on RT for 5 min.

3) 0.2 ml Chloroform was added per 1 ml of Trizol reagent used. Tubes were vigorously shaken by hand for 15-20 seconds and incubated 3 min at RT.

4) Tubes were centrifuged at 12,000 x g for 15 min at 4°C

5) The aqueous upper phase was transferred to a fresh RNase-free Low-bind (Eppendorf) tube and 0,5 ml isopropanol (per 1 ml initial of Trizol) were added, and mixed well by shaking the tube by hand.

6) Samples were incubated at RT for 10 min and centrifuged at no more than 12,000 x g for 15 min at 4°C to pellet the RNA.

7) RNA pellet was washed once with 1 ml of 75% cold EtOH, and samples were shaken and centrifuged at 8,000 x g at 4°C for 10 min.

8) Supernatant was removed as well as possible (without disturbing the RNA pellet) and pellets were air-dried (not completely to avoid a decrease in RNA solubility).

9) RNA pellet was finally dissolved in an appropriate volume of nuclease-free water (Ambion) and stored at -80°C.

Since, the amount of isolated total RNA varied among samples depending on the starting material (number of primary cultured cells), RNA quantity was evaluated by NanoDrop spectrophotometry (Thermo Scientific) and Quant-iT RNA Assay Kit on the Qubit Fluorometer (Invitrogen), with consisting results. Prior to continuing to the next step, integrity of isolated RNA was also evaluated by Experion (Biorad), following the manufacturer’s instructions. In all samples we also evaluated the RNA integrity number (RIN) and it was considered as optimal (ranging from 9.5 to 10).

**B)** Ribo-depletion. About 10-12 μg of isolated total RNA underwent two rounds of ribosomal RNA depletion by using magnetic beads (RiboMinus™ Eukaryote Kit for RNA-Seq, Invitrogen) according to the manufacturer’s protocol. More in detail:

1) About 10 μg of total isolated RNA per sample were suspended in a volume of 20 μl of nuclease-free water in a sterile, RNase-free Low-bind 1.5 ml microcentrifuge tube. To this were added 10 μl of RiboMinus™ Probe (15 pmol/μl) and 300 μl of Hybridization Buffer.

2) Tubes were incubated at 72°C for 5 min to allow complete denaturation of RNA for an efficient hybridization of RiboMinus™ probes.

3) Samples were slowly cooled up to 37°C over a period of 30 min in a termomix.

This low-cooling step is crucial in the ribo-depletion process in order to promote correct sequence-specific hybridization of the LNA-containing probes.

4) While the sample is slowly cooling, we proceeded to the preparation of RiboMinus™ Magnetic (streptavidin-coated) Beads.

5) RiboMinus™ Magnetic Beads were dissolved in its bottle by vortexing.

6) 750 μl of the beads suspension were transferred into a sterile, Rnase-free Low-bind 1.5 ml microcentrifuge tube and placed on a magnetic separator for 1 min. Supernatant was gently aspirated and discarded.

7) Beads were then dissolved by slow vortexing in 750 μl of sterile DEPC water.

8) Beads were magnetically separated and the supernatant was gently aspirated and discarded.

9) Steps 8 and 9 were repeated once.

10) Beads were then dissolved in 750 μl Hybridization Buffer and 250 μl beads were transferred to a new tube (to use them for a later step).

11) The tube with 500 μl beads was placed on a magnetic separator for 1 minute, and supernatant was gently aspirated and discarded.

12) Beads were dissolved in 200 μl Hybridization Buffer and temporary stored at 37°C until use.

13) After slow-cooling of samples from the Step 4, tubes were briefly centrifuged to collect the sample to the bottom of the tube.

14) Sample (about 330 μl) was then transferred onto the prepared RiboMinus™ Magnetic beads from Step 12 and pipetted up and down to allow a complete hybridization.

15) Tube is incubated at 37°C for 15 min and, during incubation, the tube was occasionally gently mixed. The sample was then briefly centrifuged to collect it to the bottom of the tube.

16) rRNA-probe complexes were then pelletted by placing the tube on a magnetic separator for 1 min. The supernatant contains RiboMinus™ RNA.

17) Tube with 250 μl beads from Step 7 was placed on a magnetic separator for 1 min, and supernatant was gently aspirated and discarded.

18) ~530 μl of supernatant containing RiboMinus™ RNA from Step 16 is added to this tube of beads, and the tube is incubated at 37°C for 15 min and, during incubation, the tube was occasionally gently mixed. The sample was then briefly centrifuged to collect it to the bottom of the tube.

19) The supernatant (~530 μl) containing RiboMinus™ RNA was then transferred in a new tube.

RiboMinus™ RNA was concentrated using standard ethanol precipitation that ensures the recovery of smaller (<200 nt) RNA. More in detail:

1) 1 μl glycogen (20 μg/μl), 1/10^th^ sample (eluted RNA) volume (53 μl for this protocol) of 3 M sodium acetate and 2.5X sample volumes (1,325 μl for this protocol) of 100% cold ethanol were added to RiboMinus™ RNA samples.

2) Samples were incubated at –80°C for at least 30 min, and then centrifuged for 15 min ≥12.000 × g at 4°C. Supernatant was carefully discarded without disturbing the pellet.

3) 500 μl 70% cold ethanol were added, and the tubes were centrifuged for 5 min ≥12.000 × g at 4°C. Supernatant was carefully discarded without disturbing the pellet. This step was repeated once.

4) The resulting RiboMinus™ RNA pellet was air-dried for 5-10 min and resuspended in an appropriate volume of 10–30 μl nuclease-free water.

The efficiency of ribo-depletion was evaluated on a single 20 ng aliquot of RNA by using High Sensitivity RNA kit on Experion, following the manufacturer’s instructions.

It was noted that a single round of Ribominus™ is insufficient for a good rRNA depletion, and only high-quality RNAs (with a RIN>9 as assessed through Experion) can assure high yields of ribo-depleted RNA samples to be further processed. Since RiboMinus™ is designed to hybridize with highly conserved regions of 5S, 5.8S, 18S, and 28S rRNA, and is based on an oligonucleotide probe mixture (containing 3 LNA™, Locked Nucleic Acid monomers) with 2 probes each specific for a class of rRNA, even mild or partial degradation may be responsible for an incomplete removal of rRNA.

**C)** Fragmentation of RNA. Independently on the library construction procedure, particular care should be taken to avoid complete degradation of RNA during the controlled RNA fragmentation step that represents a very critical part of the entire experimental procedure. This step is crucial to convert the RNA sample to a size appropriate for SOLiD™ System sequencing.

Fragmenting RNA, rather than DNA, has the clear advantage of reducing possible secondary structures, particularly for tRNA, miRNA and other small non-coding RNA molecules, giving rise to a major heterogeneity in coverage, leading to a more comprehensive WT analysis. The widely used methods for RNA fragmentation, which appear to give an unbiased tag representation in the libraries, mostly rely on chemical and heat fragmentation.

In this work, RNA samples were fragmented, by using controlled enzymatic hydrolysis (RNase III), which allows a greater reproducibility of the results. Since temperature, sample concentration and the time of controlled enzymatic hydrolysis are important parameters in this process, we custom-optimized and calibrated this crucial step for our sample preparation. More in detail:

1) For each sample, 100 ng of RiboMinus™ RNA were resuspended in a final volume of 8 μl.

2) 1 μl 10x RNase III Buffer (200 mM Tris acetate, pH 8.2, 500 mM potassium acetate and 150 mM magnesium acetate) and 1 μl RNase III from the SOLiD™ Whole Transcriptome Analysis Kit, were added to the reaction mix.

3) The reaction was then incubated in a thermal cycler at 37°C for 10 min.

4) Immediately after the incubation, 90 μl of cold nuclease-free water were added, and the samples placed on ice.

5) A cleanup step using RiboMinus™ Concentration Module (Invitrogen), according to the manufacturer’s protocol, was performed.

6) Fragmented RNA samples were then quantified using Quant-iT RNA Assay Kit on the Qubit Fluorometer and the appropriate size distribution of fragmented RNA was evaluated on the Experion.

**Stand-oriented cDNA library preparation**

Preserving the strandedness in the cDNA library preparation is fundamental for further data analysis as it allows to correctly assess the directionality of transcription and gene orientation, facilitating thus the detection of opposing and also overlapping transcripts. More in detail:

100 ng of the fragmented RNA samples (resuspended in 3 μl) were hybridized with 3 μl of hybridization solution in 0.2 ml PCR tubes, 2 μl of Adaptor Mix A, a set of oligonucleotides with a single-stranded degenerate sequence at one end and a defined sequence required for SOLiD System sequencing at the other end. Hybridization with Adaptor Mix A yields template for SOLiD System sequencing from the 5′ end, allowing to conserve the strandedness of cDNAs during the sequencing cycles.

Afterwards, 10 μl Ligation Buffer and 2 μl Ligation Enzyme Mix were added to 8 μl of reaction mixture. RNA ligation was performed in a thermal cycler at 16°C for 16 h.

The 20 μl Reverse transcription reaction mixture prepared on ice included:

4 μl 10X RT Buffer

2 μl dNTP Mix

13 μl nuclease-free water

1 μl ArrayScript™ Reverse Transcriptase

The RT reaction mix was incubated in a thermal cycler at 42°C for 30 min.

After a cleanup step, using the MinElute® PCR Purification Kit (Invitrogen), according to manufacturer's protocol, samples were denatured on 6% TBE-Urea gel, and gel slices containing cDNA of the desired size range of 100-200 nucleotides were excised. A total of 16 cycles of PCR amplification were then performed on excised gel slices by using AmpliTaq® DNA Polymerase in a reaction volume of 100 μl.

After the PCR, the amplified cDNA were cleaned up using the PureLink™ PCR Micro Kit. The yield of PCR products was verified by Quant-iT DNA Assay Kit, on the Qubit Fluorometer and NanoDrop spectrophotometer (Invitrogen), and size distribution of each cDNA library was evaluated on the Experion.

**Mapping strategy and data visualization**

The mapping strategy was performed independently for each sample following the pipeline in Supplementary Material, Figure S2. First, the total produced reads were filtered according to their quality values removing those with median quality value less or equal than 5. Secondly, we filtered out those reads that mapped to the adapters and to the ribosomal sequences (*Homo sapiens* 5.8S, 18S and 28S rRNA). In particular, the mapping to the adapters or to the ribosomal sequences was assessed with a read length of 50 nt allowing at most 2 mismatches and with a read length 25 nt without mismatches.

After the filtering steps, usable reads underwent a general mapping scheme using RNA-MATE software [59] version 1.1. Therefore, usable reads were cyclically mapped first to human reference genome (GRCh37, hg19, excluding chromosome Y -female samples) and, subsequently, to a custom library of exon-junction sequences containing 2010842 sequences. At the end of each cycle, reads failed to map to the genome or to the junctions library were left-end trimmed using a pre-defined lengths schema defined in the following. This genomic-junction mapping scheme was repeated for each cycle.

The custom junctions library was constructed for each cycle in the following way. Each junction consists of a 2k-nt (with 2k equal to the read length minus 10) obtained by concatenating two independent k-nt sub-sequences, i.e, the right-half side consists of the last k-nt extracted from the "donor" exon and the left half-side of the first k-nt from the "acceptor" exon. Exon sequences were derived from all RefSeq genes and, for each gene the exons were combined in all possible ways (preserving the longitudinal order along the genome) in order to provide either all the annotated RefSeq junctions and combinatorial junctions. Junctions derived from Y chromosome were removed from the library (female samples).

Each junction was also classified as 1) RefSeq, 2) UCSC and 3) Ensembl junction (according to its presence in the related database) or 4) putative novel junction (for those originated from computationally-driven combinatorial process) and were unambiguously associated to the corresponding RefSeq gene.

We performed the following mapping cycles: 50.5.1, 45.4.1, 40.4.1, 35.3.1, 30.3.1, 25.1.1, 23.0.0, 22.0.0, 21.0.0, where the cycle x.y.z stands for reads of length x that are mapped with at most y mismatches, while parameter z=1 denotes that two valid adjacent mismatches are counted as a single mismatch (z=0 denotes that two valid adjacent mismatches are counted as two mismatches). The read length scheme was tuned by monitoring the percentage of antisense hits observed on the junction library (much less than 0.1% at each cycle).

RNA-MATE allows to directly assign multiple reads with a single “best hit” to that specific position; all remaining multiple reads underwent the rescue procedure with default parameters.

At the end of the alignment procedure three types of reads were identified: UARs (that includes uniquely mapped reads, reads with a unique best score - and hence uniquely truly aligned - and multiple reads that were uniquely located after the rescue procedure), MRs (multiple reads that could not be assigned by the rescue procedure) and reads without a specific mapping location (denoted as unmatched reads). All the MRs were discarded from further analysis, while UARs and reads mapping to junctions were used for signal quantification.

For each sample, the result of the mapping strategy consists of strand specific wiggle files (containing the number of reads covering each base of the genome), of a couple of bed files containing the number of hits per each junction in the database (expected junctions strand specific, unexpected junction antisense) and strand specific of starts files containing the starting position of each mapped (and uniquely placed) read. Such files were visualized in UCSC Genome Browser [63] to assess the quality and the consistency of the mapping. Additionally, a specific pipeline was built for converting the standard RNA-mate output in the SAM/BAM format.

**Annotation files and quantitative measures**

As a reference for the quantitative estimation of gene expressions in both trisomic and euploid cells, we have used ReqSeq gene model annotation downloaded from UCSC genome browser [62]. However, since the RefSeq track coordinates are generated by aligning the RefSeq sequences to genome with the BLAT tool [82], it is possible that transcripts with highly similar sequences to each other are assigned to multiple locations. This leads to ambiguity in the localization of individual transcripts, but it is important to take them into account, because it is likely that the reads sequenced from transcripts in this condition will be MRs.

To avoid mis-specifications or ambiguities due to the overlap of multiple transcripts in the same genomic position (i.e., same locus and same strand) we first identified and grouped together any transcript with intersecting coordinates at the exon level (if one transcript was entirely included into the intron on another gene they were considered as non-overlapping).

We then decided to separate the cases with a reciprocal overlap > 50 bp (at least the length of a read), although classifying them separately (when present, in each of the classes described below) to take track of them. We classified as “Lonely” (14809) and “SmallOverlap50bp” (40) the non-overlapping transcripts and then used the associated gene symbol to further classify all the groups. To reduce mis-annotation errors, along with the gene symbol provided by UCSC, we also considered the gene to RefSeq association downloaded from Entrez Gene database [83] and took as synonymous all the symbols associated to each transcript. Using this information, we classified as: “Onegene” (5755) or “Onegene_NoSmall” (10) the groups in which all members had the same gene symbol; “GeneMultiloci” (52) the groups in which a gene was annotated to have multiple loci; “GeneSynonym” (92) the groups in which all members shared at least one common synomymous; “Family” (218) the cases in which all the members had symbols only differing in the last part of the gene (i.e. “GENE1”, “GENE13”, etc.); “HighOverlap50” (32) all the groups in which all the transcripts share more than 50% of the coding sequence and “ExonSharing” (114) all the remaining cases. We note that the majority of transcripts have only one form and the number of ambiguous cases is relatively small since RefSeq is a curate and conservative source of annotation, in which purely putative transcripts are not present.

After producing this classification, within each group we fused all the coordinates of the members together and split the partially overlapping exons in order to have only disjoint elements, each of them could be mapped back to all the transcripts containing it. At the end, we produced a modified RefSeq based annotation file containing 215952 annotated elements (i.e., exons or part of exons) in a BED format corresponding to 21122 uniquely identified (and non redundant) RefSeq genes or group/family of RefSeq genes (The revised annotation file is available upon request).

A preliminary estimate of the global expression was obtained by computing the number of UARs starting in all the annotated elements (i.e., exons or part of exons) corresponding to the same gene in the annotation. In particular, for genes with a single annotated transcript such measure corresponds to the number of UARs mapping to the entire length of the transcript, whilst, for genes with multiple splice isoforms, it corresponds to a global activity estimated summing together all the reads mapped to any independent exon of each possible transcripts. In both cases to reduce the bias, the final expression value was corrected by adding to each specific locus the read counts derived from the splice junctions.

Finally, to account for transcripts of different lengths, the gene expression values of annotated loci were also expressed as reads per kb of transcript (or gene model) per million mapped reads (RPKM) and for each only *loci* with RMKM >0.1 in at least one sample were considered active.

Expressed genes were further classified for both samples according to RPKM values in 5 categories: 1) very low expression (i.e., RPKM range 0.1-1), 2) low expression (i.e., RPKM range 1-5), 3) intermediate (i.e., RPKM range 5-50), 4) high (i.e., RPKM range 50-500) and 5) very high expression (i.e., RPKM greater than 500).

The analysis of RefSeq loci was also aimed to detect a particular enrichment in 3' (or 5') UTRs. To this purpose, for each annotated RefSeq gene, we considered a window of 150bp extending 3' (or 5') UTRs and we computed the observed coverage in each of such windows.

**Identification of alternative splicing events**

We inferred the evidence of multiple isoforms on the basis of the reads mapping to the splicing junctions. In particular, we considered as alternative splicing markers either the multiple donors or the multiple acceptor (or both) junctions (i.e., “guilty-by-evidence” approach).

Junctions multiple donors were defined as junctions sequence with the same right side (i.e., extracted from the same position of the genome) and different left side, *viceversa* junctions multiple acceptors were defined as junctions sequence with the same left side and different right side.

A junction was considered reliable if there were at least T1 reads mapped on it (in principle more sophisticated quality measures like the one proposed in [77], can be used in a similar way).

Then the identification proceeded as follows. First, for each sample, we retrieved all the reliable junctions and, among them we selected those containing either multiple donors or multiple acceptors. Then, RefSeq genes containing such junctions were detected. Such genes constitute a preliminary list of candidates to the evidence of multiple splicing isoforms. To remove mapping artefacts this lists can be further filtered using information arising from exon by exon map usage.

Secondly, the two samples were cross-compared as follows: the splice junctions common to both samples were identified and, for each sample, two lists of candidates (sample specific junctions) were obtained. To remove the effect of the user specific threshold T1 in the selection, each list was subsequently filtered removing those junctions that received any number of hits in the other sample. In practice, the resulting sample specific alternative splicing junctions were characterized as being reliable multiple donor or multiple junctions in one sample that did not received any hits in the other sample.

Finally, since each junction was also classified as RefSeq junction, UCSC junction, Ensembl junction or as putative new junction accordingly to if it was annotated in the corresponding database or if it was a results of a pure combinatorial process. Junctions’ classification was used to detect those genes containing putative new junctions that are candidate to reveal the presence of novel (un-annotated) isoforms.

**Refinement of non-RefSeq loci**

From the RefSeq annotation files we also defined for each strand the intergenic and intronic regions (igRs and inRs, respectively). ChrM was also independently analyzed.

The inRs were defined as the genomic regions separating two consecutive non-overlapping RefSeq genes provided that no other RefSeq loci mapped across this region, whilst igRs were defined as the genomic regions separating two consecutive exons of the same RefSeq gene (independently from the isoform) or two consecutive exons in overlapping RefSeq genes. The annotation was performed independently on each strand, the regions were labelled, enumerated and stored in a BED file for visualization purposes.

Each region was independently quantified in each sample to provide a measure of the overall mapping in non-RefSeq regions.

Strikingly, we noted that igRs and inRs accounted for about 40% of the total mapped reads, although the length of such regions was much higher than RefSeq annotated loci (considering both strands of the human genome about 4.9x10^9^ and 1x10^9^, respectively).

Therefore, to measure the signal strength in yet unannotated regions, both igRs and inRs were filtered on the basis of the UCSC and Ensembl annotation (i.e., the base positions annotated in UCSC and Ensembl databases as genes were removed and the regions were subdivided, if necessary). Remaining regions were re-labelled, enumerated and the read counting procedure was repeated for both samples. Such filtering allowed to remove ambiguities due to annotation database, but had a negligible impact on the size of the redefined igRs and inRs.

Subsequently, each unannotated genomic region (redefined igRs and inRs) showing the presence of signal (i.e., mapped reads) underwent a data-driven refinement procedure aimed to more precisely define the location of small active strand-specific subregions (igTARs and inTARs). IgTARs and inTARs will consist in reads-dense subregions within igRs and inRs respectively.

The data-driven refinement procedure mimicked the bisection approach and proceeded as follows:

- Each region larger than a pre-assigned windows W and containing more than T mapped reads was divided in two equal subregions and the read count was repeated in each part;

- Regions larger than W and containing less then T reads were removed from the analysis and labelled as “low count” regions;

- Regions smaller than W and containing more than T reads were stored and labelled as “interesting regions”.

The splitting process was iteratively repeated until no more regions were to split, and all the initial regions were classified either as “interesting” or “low count” regions. Then, all interesting regions were retrieved, while low count ones were retrieved only if their actual reads count/W ratio was higher than 1.5; remaining regions were discarded from further analyses. Finally, contiguous retrieved subregions were merged in a single larger region (either igTARs or inTARs). Identified igTARs and inTARs were then labelled, enumerated and stored in a BED format for quantification purposes.

IgTARs and inTARs constitute a first approximate localization of where the signal is. It's clear that dyadic subdivision of the igRs and inRs is suboptimal; however, the precise boundaries of igTARs and inTARs can be further characterized using simple translations, dilatations and contractions from the previously estimated igTARs and inTARs.

**Statistical tests for differential expression**

In order to detect differential expression between trisomic and euploid states, we first compared the two states at RefSeq level, then we considered the newly defined unannotated IgTAR and InTAR. Statistical significance has been inferred from the observed total reads count (UARs + reads from junctions) in each locus using a combination of tests, namely DEGseq [77], DESeq [78] and edgeR [79] for which suitable R-package are available under Bioconductor (www.bioconductor.org/packages/2.7).

Such tests are based on slightly different assumptions that usually produce different level of stringency - and sometime different results - in particular when applied to small sample experiments. However, all of them appear to be particularly suited for RNA-Seq data, hence they were independently applied to the analysis of the two states with the below described choice of parameters.

The results of each selection were also cross-compared either to compromise with their assumptions and to illustrate the impact of them in the final choice.

As a general remark we noticed that all tests assume that the observed data are counts, hence they are discrete and skewed (not well approximated by a normal distribution). To model observed counts Poisson or negative binomial distribution are usually considered.

Both DESeq and EdgeR assume that gene counts are distributed accordingly with negative binomial. They mainly differ on the way they estimate the extra term in the variance that is used to model the over-dispersion observed in biological replicates.

Indeed, due to the lack of biological replicates, edgeR measures the over-dispersion using common parameters, whilst DESeq estimates the variance in a local fashion using a regression approach. Vice versa, DEGseq, with the Fisher Test method, implicitly assumes the Poisson distribution on the observed counts.

All methods were applied to the observed reads counts in both to DS and Euploid state, either using their internal normalization procedure and a specific normalization procedure based on the total read mapped in each sample. Clearly, in the latter case, when considering the total reads mapped in each sample, we removed the reads that mapped on chromosome 21 - due to the trisomy the fraction of mapping in DS is observed and expected higher than in Euploid - and ones the mitochondrial chromosome.

For each RefSeq gene we compute the p-value and its corresponding adjusted p-value or q-value that was used to detect significant changes in expression. A cut-off of 0.1 was used for DESeq (that resulted very conservative for small samples), while a cut off of 0.0001 was used for both edgeR and DEGseq (both of them resulted to be quite permissive).

In particular, observing the distribution of genes detected as DE in Figure 6, we noticed that most of the genes detected by edgeR and DEGseq consists in highly expressed genes a with relatively small difference between the two states. Such situation seems more likely to be due to an underestimate of the variance term rather than a true biological difference. Therefore, a threshold of 1.5 on the fold change between the normalized samples was imposed to filter out those genes whose significance appeared marginal.

Each evidence of DE was finally classified as strong (i.e., selected by all three methods), good (i.e., selected by two out of three methods), and acceptable (i.e., selected by only one method out of three). All DE genes below the fold-change threshold, but significant in at least one test, were classified as weak.

**GO analysis**

Since in RNA-Seq experiments longer and more highly expressed transcripts are likely to have a greater number of reads and more statistical power for detecting DE between samples, widely used tools for gene ontology (GO) analysis are biased towards detecting DE genes with a high number of reads [84]. To avoid this bias, we used a very recently developed statistical methodology, namely GO-Seq [71] that allows an unbiased GO analysis for large-scale RNA-Seq data.

In order to identify the enriched GO terms associated to DE genes, we considered in the analysis only the RefSeq associated to unique Entrez Gene Ids (thus discarding all the heterogeneous groups or families) and the analysis was performed using standard parameters (Wallenius approximation and adjusted p-value threshold 0.05).

**References in File S1**

12. Costa V, Sommese L, Casamassimi A, Colicchio R, Angelini C, et al. (2010) Impairment of circulating endothelial progenitors in Down syndrome. BMC Med Genomics 3: 40.

58. Casamassimi A, Balestrieri ML, Fiorito C, Schiano C, Maione C, et al. (2007) Comparison between total endothelial progenitor cell isolation versus enriched CD133+ culture. J Biochem 141: 503-511.

59. Cloonan N, Xu Q, Faulkner GJ, Taylor DF, Tang DT, et al. (2009) RNA-MATE: a recursive mapping strategy for high-throughput RNAsequencing data. Bioinformatics 25: 2615–2616.

62. Kuhn RM, Karolchik D, Zweig AS, Wang T, Smith KE, et al. (2009) The UCSC Genome Browser Database: update 2009. Nucleic Acids Res 37(Database issue): D755-761.

63. Hsu F, Kent WJ, Clawson H, Kuhn RM, Diekhans M, et al. (2006) The UCSC Known Genes. Bioinformatics 22: 1036-1046.

71. Young MD, Wakefield MJ, Smyth GK, Oshlack A (2010) Gene ontology analysis for RNA-seq: accounting for selection bias. Genome Biol 11: R14.

77. Gremmels H, Fledderus JO, Balkom BW, Verhaar MC (2011) Transcriptome Analysis in Endothelial Progenitor Cell Biology. Antioxid Redox Signal. Feb 14.

78. Livak KJ, Schmittgen TD (2001) Analysis of relative gene expression data using real-time quantitative PCR and the 2(-Delta Delta C(T)) Method. Methods 25: 402-408.

79. Wang L, Feng Z, Wang X, Wang X, Zhang X, (2010) DEGseq: an R package for identifying differentially expressed genes from RNA-seq data. Bioinformatics 26: 136-138.

82. Kent WJ (2002) BLAT - the BLAST-like alignment tool. Genome Res 12: 656-664.

83. Maglott D, Ostell J, Pruitt KD, Tatusova T (2005) Entrez Gene: gene-centered information at NCBI. Nucleic Acids Res 33(Database issue): D54-58.

84. Oshlack A, Wakefield MJ (2009) Transcript length bias in RNA-seq data confounds systems biology. Biol Direct 4: 14.
